# A hierarchical stochastic model for bistable perception

**DOI:** 10.1371/journal.pcbi.1005856

**Published:** 2017-11-20

**Authors:** Stefan Albert, Katharina Schmack, Philipp Sterzer, Gaby Schneider

**Affiliations:** 1 Institute of Mathematics, Goethe University, Frankfurt (Main), Germany; 2 Department of Psychiatry and Psychotherapy, Charité Universitätsmedizin Berlin, Germany; Technische Universitat Chemnitz, GERMANY

## Abstract

Viewing of ambiguous stimuli can lead to bistable perception alternating between the possible percepts. During continuous presentation of ambiguous stimuli, percept changes occur as single events, whereas during intermittent presentation of ambiguous stimuli, percept changes occur at more or less regular intervals either as single events or bursts. Response patterns can be highly variable and have been reported to show systematic differences between patients with schizophrenia and healthy controls. Existing models of bistable perception often use detailed assumptions and large parameter sets which make parameter estimation challenging. Here we propose a parsimonious stochastic model that provides a link between empirical data analysis of the observed response patterns and detailed models of underlying neuronal processes. Firstly, we use a Hidden Markov Model (HMM) for the times between percept changes, which assumes one single state in continuous presentation and a stable and an unstable state in intermittent presentation. The HMM captures the observed differences between patients with schizophrenia and healthy controls, but remains descriptive. Therefore, we secondly propose a hierarchical Brownian model (HBM), which produces similar response patterns but also provides a relation to potential underlying mechanisms. The main idea is that neuronal activity is described as an activity difference between two competing neuronal populations reflected in Brownian motions with drift. This differential activity generates switching between the two conflicting percepts and between stable and unstable states with similar mechanisms on different neuronal levels. With only a small number of parameters, the HBM can be fitted closely to a high variety of response patterns and captures group differences between healthy controls and patients with schizophrenia. At the same time, it provides a link to mechanistic models of bistable perception, linking the group differences to potential underlying mechanisms.

## Author summary

Patients suffering from schizophrenia show specific cognitive deficits. One way to study these cognitive phenomena works with the presentation of ambiguous stimuli. During viewing of an ambiguous stimulus, perception alters spontaneously between different percepts. Percept changes occur as single events during continuous presentation, whereas during intermittent presentation, percept changes occur at regular intervals either as single events or bursts. Here we investigate perceptual responses to continuous and intermittent stimulation in healthy control subjects and patients with schizophrenia. Interestingly, the response patterns can be highly variable but show systematic group differences. We propose a model that connects these perceptual responses to underlying neuronal processes. The model mainly describes the activity difference between competing neuronal populations on different neuronal levels. In a hierarchical manner, the differential activity generates switching between the conflicting percepts as well as between states of higher and lower perceptual stability. By fitting the model directly to empirical responses, a high variety of patterns can be reproduced, and group differences between healthy controls and patients with schizophrenia can be captured. This helps to link the observed group differences to potential neuronal mechanisms, suggesting that patients with schizophrenia tend to spend more time in neuronal states of lower perceptual stability.

## Introduction

The phenomenon of bistable perception has fascinated researchers for a long time [1, 2, 3]. Recently, the description of response patterns to bistable stimuli such as the Necker Cube, Rubin’s vase or rotating spheres with switching rotation direction gained increasing interest in computational neuroscience [4, 5, 6, 7, 8]. By modeling dynamic changes of perception during viewing of one and the same stimulus, one aims at providing potential explanations for neuronal mechanisms underlying perception and perceptual changes and to identify related brain areas as well as potential dysfunctions, e.g. in schizophrenia [9, 10].

Interestingly, the response patterns to continuously shown bistable stimuli often share common properties [7, 11]. Typically, the distribution of intervals of constant perception, termed dominance times, is unimodal and right-skewed, and extremely short dominance times, i.e., rapidly fluctuating precepts, are rare [12, 13, 14]. The dominance times under continuous stimulation are therefore often modeled as Gamma distributed [15, 16, 17, 18, 19, 20]. The mean of dominance times can be highly variable across subjects [14, 20], whereas the coefficient of variation (CV) is often comparable [21].

**Fig 1:**
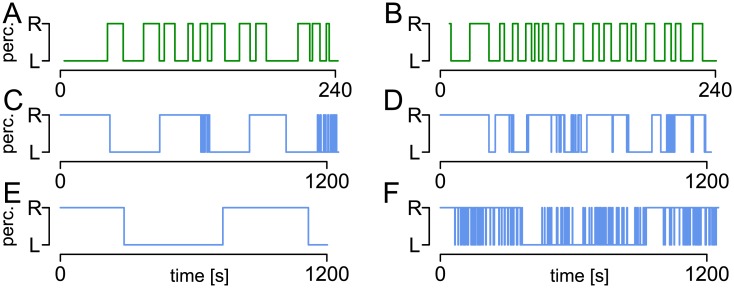
Examples of response patterns to a bistable stimulus. Response patterns to continuous (green, A,B) and intermittent (blue, C-F) presentation from the dataset reported in [10]. While the distribution of dominance times tends to be unimodal in the continuous case, stable and unstable phases interchange in intermittent stimulation. In addition, response patterns can be highly variable across subjects.

In comparison to a continuous presentation, intermittent presentation of a bistable stimulus, i.e., by repetitive interruption of stimulation for short time periods, has been observed to stabilize the percept if the interruption period is long enough, typically longer than 0.7 seconds [15, 18, 22, 23, 24, 25]. In this case, dominance times get longer and can also show a certain degree of periodicity [14]. In addition, such stable phases with long dominance times during intermittent presentation can also interchange with unstable phases of rapid percept changes. Fig 1 shows examples of response patterns to continuous and intermittent presentation of a bistable stimulus from the dataset reported in [10].

Modeling studies with elaborated mathematical models have been proposed that can explain a number of properties of bistable perception like the distribution of dominance times under continuous stimulation [8, 17, 19, 20, 21, 26] or cyclic behavior and the impact of the duration of the stimulus presentation on the dominance times in intermittent stimulation [14, 18]. One key ingredient of these models of bistable perception is typically a competition between neuronal populations that correspond to the different percepts [14, 17, 18, 20, 27]. In order to account for stabilized perception in intermittent viewing, the use of multiple timescales for memory traces of past perception has been proposed by [14] and [18].

Many such models require a high number of parameters in order to describe the variety of response patterns. As a consequence, they can often hardly be fitted to experimental data, especially in the typical cases when only a few dozen dominance times are observed. In addition, the majority of models focus either on continuous or on intermittent viewing. Interesting models that are applicable to both cases have been proposed by [14, 17, 18].

**Fig 2:**
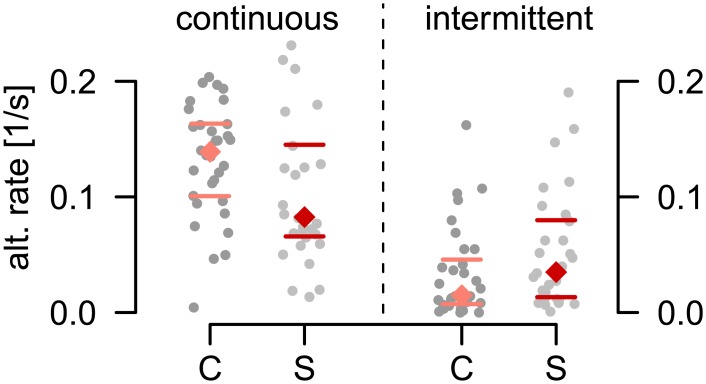
Alternation rates in control subjects and subjects with schizophrenia. During continuous presentation healthy controls (C) showed higher alternation rates compared to patients with schizophrenia (S) (left), while the opposite could be observed for intermittent presentation (right). Each grey dot indicates the perceptual alternation rate from one individual participant, colored diamonds and lines indicate group medians with 25%/75% quantiles. Two-sided Wilcoxon tests yielded *p* < .1 for both continuous and intermittent stimulation.

The relevance of a joint description of continuous and intermittent viewing is illustrated here on a dataset including responses of patients with schizophrenia and of healthy controls to continuous and intermittent presentation of a rotating sphere with ambiguous rotation direction reported earlier in [9, 10]. In [10], an enhanced alternation rate for the group of patients with schizophrenia during intermittent presentation was reported. Interestingly, when we analyzed previously unpublished data recorded in the same participants during continuous presentation, the opposite could be observed [Fig 2; the data was collected during an initial training run for which the experimental procedures but not the results are described in 10]. Due to the differences in patterns and time scales between continuous and intermittent presentation, the potential neuronal mechanisms underlying the transitions between the different response properties remain unclear.

Therefore, we propose here a new model for the description of response patterns to bistable perception that links the observed behavior in continuous and intermittent stimulation to potential underlying neuronal processes. First, the model should be able to describe the high variety of both, continuous and intermittent stimulation within one model framework. Second, we use a minimal number of parameters in order to allow parameter estimation and model fitting to the typically short experimental data. This can then allow the statistical investigation of differences between clinical groups.

Note that strictly speaking, the term ‘dominance time’ refers to slightly different objects in continuous and intermittent viewing. While during continuous presentation, switches occur from a dominant to a suppressed percept (percept-switch), dominance times during intermittent presentation consist of multiple continuous presentation periods, and switches typically occur because of different perceptual choices at the onset of the presentation (percept-choice) [28]. In the present model, the observed sequences of dominance times are treated as conceptually similar. This simplification allows for a parsimonious model description in both continuous and intermittent viewing but may not fully capture the relation between the perceptual processes in the two regimes.

The remainder of the article is organized as follows. First, we use a simple Hidden Markov Model (HMM) that describes the observed perceptual processes with a few parameters. For continuous presentation, one state produces independent and identically distributed dominance times with a two-parametric distribution. For intermittent presentation, switching between stable and unstable phases requires two hidden states with short and long dominance times, respectively. The HMM has the advantage that it allows straightforward model fitting and data description with a minimal number of parameters. However, it remains descriptive and lacks relations to potential underlying mechanisms. Therefore, we link the HMM to a hypothetical underlying stochastic model. This model is termed here Hierarchical Brownian Model (HBM) and intends to describe aggregated underlying neuronal activity, producing the observed behavioral responses.

The HBM is based on two main ideas: First, it assumes that switching between percepts results from two conflicting neuronal populations [cmp., e.g., 18]. In order to minimize the number of parameters, this process is reduced to a simple Brownian motion with drift that fluctuates between two thresholds, where the first passage times indicate state changes [similar to 21]. For continuous presentation, one therefore requires only two parameters, i.e., the drift of the Brownian motion and the threshold. The distribution of the resulting first passage times—i.e., dominance times—is then the same as in the HMM, with a simple relation between the two HBM and the two HMM parameters. Second, in order to describe intermittent presentation in the same model framework, we use a hierarchical model. The idea is to describe the switching between stable and unstable phases that is typical for intermittent presentation by using an analogous threshold crossing mechanism of conflicting neuronal populations. Specifically, we assume a second pair of neuronal populations whose corresponding Brownian motion modulates the drift and threshold of the first population pair and thus causes switching between stable and unstable phases. We give a set of model assumptions under which the HBM parameters are comparable to the HMM parameters, thus allowing both model fitting to experimental data sets and potential relation to underlying mechanisms. The parameter estimation is straightforward using maximum likelihood and the HBM can reproduce both, the unimodal distribution in the continuous presentation and the bimodal distribution of dominance times in the intermittent presentation, including also various different response patterns. Moreover, it allows the identification of specific differences between the clinical groups in [10] and relates these to the hypothesized underlying processes.

## Results

### A simple Hidden Markov Model for perceptual responses

As a first step to describe the processes observed in bistable perception, we reduce data analysis to the dominance times, i.e., the times between reported changes of the percept. As described above, the distribution of dominance times tends to be unimodal in the continuous case, while stable and unstable phases interchange in intermittent stimulation.

**Fig 3:**
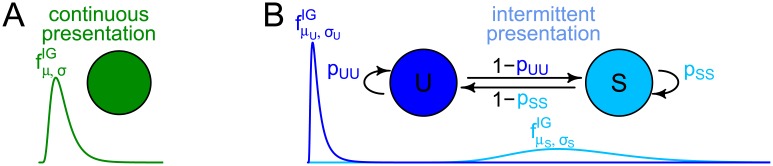
A simple HMM for bistable perception. (A) One state describes a unimodal distribution of dominance times under continuous presentation. (B) Two states (stable, S, and unstable, U) produce long and short dominance times under intermittent presentation.

We denote the dominance times by *d*_*i*_, *i* = 1, 2, …, *n*. In the unimodal continuous case, we assume the *d*_*i*_ to be the realizations of independent and identically (i.i.d.) distributed random variables *D*_*i*_, *i* = 1, 2, …, *n* (Fig 3A), where the Gamma or the Inverse Gaussian (IG) distribution are suitable two-parametric distributions [17, 18, 19, 21]. For comparability with the HBM, we focus on the Inverse Gaussian distribution here. For the intermittent case, we assume a HMM with a stable and an unstable state, which are hidden and produce long and short dominance times, respectively (Fig 3B). This requires two parameters for the switches between states, and two parameters for the distribution of dominance times in each state.

Formally, let *Y* ≔ (*Y*_*i*_)_*i* = 1,…,*n*_ describe a Markov chain on {*S*, *U*}, where *S* and *U* denote the stable and unstable state, respectively. Let *p*_*SU*_ = 1 − *p*_*SS*_ and *p*_*US*_ = 1 − *p*_*UU*_ denote the transition probabilities. The dominance times (*d*_*i*_)_*i*∈{1,2,…,*n*}_ are assumed to be Inverse Gaussian distributed and conditionally independent given *Y*, with mean and standard deviation given by (*μ*_*S*_, *σ*_*S*_) for *Y*_*i*_ = *S* and (*μ*_*U*_, *σ*_*U*_) for *Y*_*i*_ = *U*.

Note that independence of dominance times is assumed here in continuous presentation. This assumption enables straightforward parameter estimation and is in agreement with the observation that serial correlations of dominance times are typically not reported [e.g., 29, 30]. However, weak long-term dependencies of dominance times reported under continuous presentation [31] cannot be reproduced in the HMM. As such long-term dependence was not observed in the majority of cases in the present data set, also showing no group differences, we use here the simple assumption of independence. In addition, the two used HMM parameters are sufficient to capture the main group difference in the response properties reflected in the alternation rate.

#### Parameter estimation and precision of parameter estimates

*Continuous presentation*. For parameter estimation in the continuous case, we simply estimate the parameters of the Inverse Gaussian distribution from the dominance times *d*_1_, …, *d*_*n*_, assuming these are realizations of i.i.d. random variables. Let

$${\bar{\mu }}_{d}\;≔\;\frac{1}{n}\;\sum_{i=1}^{n}\;d_{i}$$

denote the sample mean of the observed dominance times. For the Inverse Gaussian (IG) distribution [32] with mean *μ* and standard deviation *σ*, the maximum likelihood (ML) estimators are given by

$$\begin{array}{c} \hat{\mu }={\bar{\mu }}_{d}\;\text{and}\;\hat{\sigma }=\sqrt{{\bar{\mu }}_{d}^{3}\frac{1}{n}\;\sum_{i=1}^{n}\;(\frac{1}{d_{i}}-\frac{1}{{\bar{\mu }}_{d}})}. \end{array}$$ (1)

**Fig 4:**
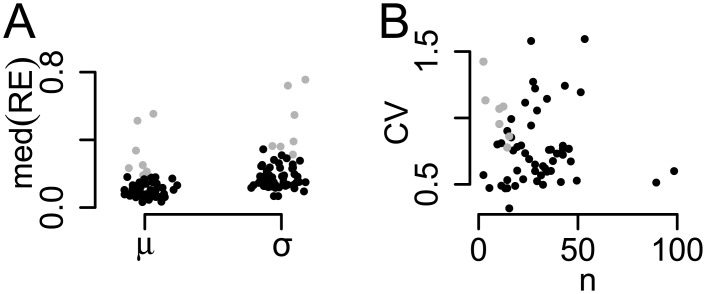
Precision of parameter estimates in the one-state HMM. For each of 61 parameter constellations in continuous presentation, 1000 simulations were performed with sample sizes as in the original data. (A) Median of the relative error (RE) for each parameter. (B) The CV as a function of the number *n* of simulated dominance times. Black points indicate constellations with mean RE across the parameters smaller than 0.25.

The precision of these ML estimators is investigated by parametric bootstrap for the parameter values estimated from the 61 response patterns to continuous presentation in the sample dataset of [10]. For each parameter combination (*μ*_*i*_, *σ*_*i*_)_*i* = 1,…,61_ we simulated 1000 response patterns with length *T* = 240 as in the original data. We then compared the estimators ${\left({\hat{\mu }}_{i},{\hat{\sigma }}_{i}\right)}_{i=1,\ldots ,61}$ with the parameter values underlying the simulation using the relative error (RE), defined e.g. for *μ* as $\text{RE}\left(\mu \right)\;≔\;|\hat{\mu }-\mu |/\mu$. The median relative errors for the 61 parameter constellations are shown in Fig 4A. Out of these, 54 (89%) showed estimation errors with median REs less than 0.25 (across the two parameters *μ* and *σ*, black). The remaining simulations (gray) showed only few percept changes, *n* < 20, as well as large CVs (*σ*/*μ*, Fig 4B).

*Intermittent presentation*. In order to estimate the parameters in the two-state HMM in intermittent presentation, we use the Baum-Welch-Alghorithm [BWA, 33, 34, 35]. See section ‘Intermittent presentation: Baum-Welch-Algorithm’ in the Materials and methods for details on the BWA, the choice of starting values and specifications in the sample data set. For details on HMMs see, e.g., [36, 37].

**Fig 5:**
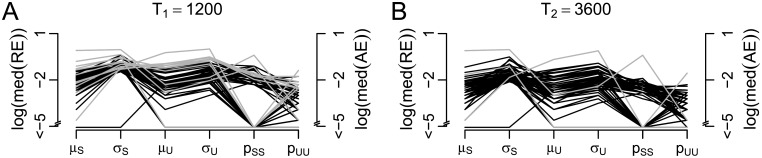
Precision of parameter estimates in the two-state HMM. For each of 61 parameter constellations in intermittent presentation, 1000 simulations were performed. Log(median) of RE (for *μ*_*S*_, *σ*_*S*_, *μ*_*U*_, *σ*_*U*_) or of AE (for *p*_*SS*_, *p*_*UU*_) for *T*_1_ = 1200 (A) and *T*_2_ = 3600 (B). Black lines indicate constellations with mean errors across the parameters <0.25.

In order to investigate the estimation precision of this approach, we again apply parametric bootstrap to the 61 parameter combinations estimated from the response patterns to intermittent presentation in the sample data set. Fig 5 shows the median errors obtained in 1000 simulations for every parameter constellation for *T*_1_ = 1200 s and *T*_2_ = 3600 s. For *p*_*SS*_ and *p*_*UU*_ the absolute errors (i.e., $\text{AE}\left(p_{SS}\right)\;≔\;\left|{\hat{p}}_{SS}-p_{SS}\right|$) are presented due to the small values of the two parameters. For the time horizon of the data, *T*_1_ (panel A), 50 of the 61 parameter combinations yielded average errors (i.e. mean median errors across all parameters) smaller than 0.25 (black). The remaining cases (gray) showed a large *μ*_*U*_, i.e., less distinguishable stable and unstable distribution, or small sample size *n* ≈ 10. For the larger time horizon *T*_2_ (panel B), almost all parameter combinations showed errors smaller than 0.25.

#### Application of the HMM to the sample data set

Here we use the described methods in order to apply the HMM to the sample dataset presented in [10] consisting of responses to continuous and intermittent stimulation obtained from each of 29 patients with schizophrenia and 32 healthy controls (for details on experimental procedures see Materials and methods). While response patterns were highly variable across subjects, the CV of dominance times (mean 0.79, SEM 0.04) was comparable to other studies reported in [21]. Serial correlation of adjacent dominance times of the same percept was typically small (mean of Kendall’s rank correlation $\bar{\tau }=0.02$), and statistically significant on the 5% level in less than 7% of the cases, which is about chance level. Concerning long-term dependence [cmp. 31], deviations from the assumption of independent dominance times were not observed in 81% of the cases, and no differences were observed between the experimental groups (*p* > .1, Wilcoxon test, for details on the analysis see Materials and methods). A correlation between the alternation rates in continuous and intermittent stimulation across subjects was not observed in either group, comparable to the results of [14].

**Fig 6:**
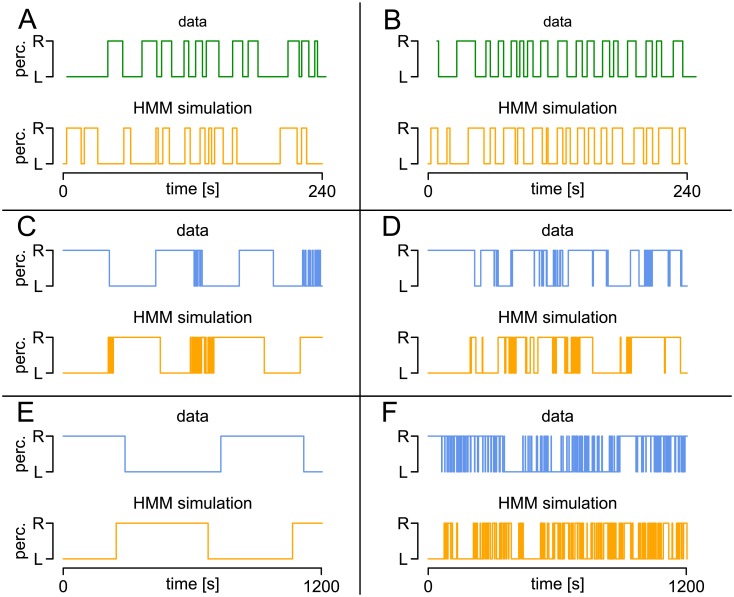
Comparison of empirical response patterns to patterns simulated with the HMM. Examples of response patterns to continuous (green, panels A and B) and intermittent (blue, C-F) stimulation repeated from Fig 1 and corresponding simulations within the HMM (orange). The parameter estimates are given in Tables 1 and 2, respectively.

*Model fit*. By fitting the HMM to response patterns in continuous and intermittent presentation as described above, the typical properties of the observed response patterns can be reproduced in simulations, including unimodal distributions for continuous presentation and changes between stable and unstable stages in intermittent presentation and a high variety of response patterns (Fig 6). For example, subject C shows rather regular stable phases, separated by unstable phases, while subject D shows an irregular response pattern, subject E shows only stable phases, while subject F shows almost only unstable phases. The parameter estimates of these example subjects are given in Tables 1 and 2. Note also that the response patterns of seven of the 61 subjects were described better by only one (stable or unstable) distribution than by the two-state HMM.

**Table 1:** Estimated HMM parameters of the response pattens to continuous presentation shown in Fig 1A and 1B.

com.	$\hat{\mu }$	$\hat{\sigma }$
A	10.50	8.18
B	6.69	3.58

**Table 2:** Estimated HMM parameters of the response pattens to intermittent presentation shown in Fig 1C–1F.

com.	${\hat{\mu }}_{S}$	${\hat{\sigma }}_{S}$	${\hat{\mu }}_{U}$	${\hat{\sigma }}_{U}$	${\hat{p}}_{SS}$	${\hat{p}}_{UU}$
C	186.45	30.50	5.01	3.06	0.67	0.96
D	67.12	50.81	3.25	2.76	0.33	0.82
E	372.28	68.30	NA	NA	1.00	NA
F	77.13	9.99	5.37	6.26	0.00	0.99

**Fig 7:**
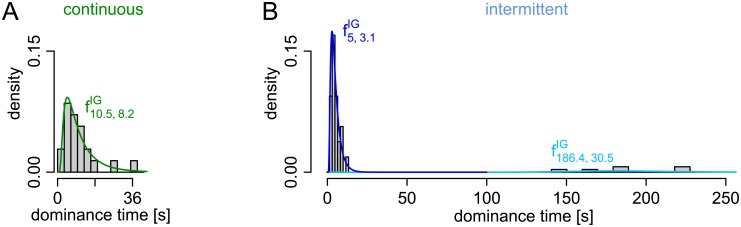
Comparison of distribution of dominance times with theoretical distribution. The theoretical IG distribution in the HMM fitted to the empirical distribution of dominance times for continuous (A) and intermittent (B) presentation shown in Fig 6A and 6C.

In addition to the good correspondence in the response patterns, no strong deviations could be observed from the model assumption of Inverse Gaussian distributed dominance times (Fig 7).

**Fig 8:**
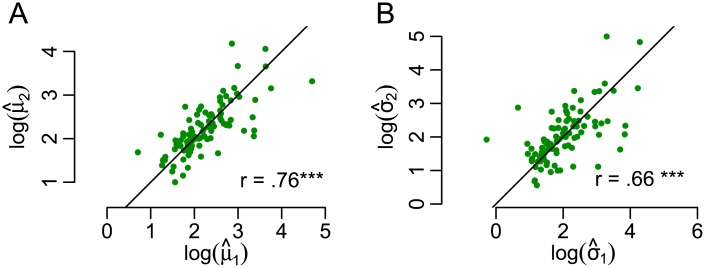
Reproducibility of response patterns. Parameter estimates of log(*μ*) (A) and log(*σ*) (B) of the IG distribution in two sessions with the same individuals, dataset reported in [9]. The logarithm was applied due to asymmetric distributions of the parameter estimates. Stars indicate highly significant (*p* < .0001) correlation of parameter estimates across different sessions.

*Comparison of repeated trials*. The HMM approach also allows studying the reproducibility of response parameters of subjects across multiple sessions. To this end, we used an additional dataset from 105 healthy individuals that contained two separate sessions of continuous presentation [see 9, here we used the first two training runs from Behavioral Experiment 2 as described in this previous work]. These showed highly reproducible response patterns, i.e., a high correlation of parameter estimates of the IG distribution of the same individuals across different sessions (Fig 8).

In addition, we also used a likelihood ratio test [38] to investigate the null hypothesis of equality of the parameters of two Inverse Gaussian distributed samples with sample sizes *n*_1_ and *n*_2_, i.e. *H*_0_: *μ*_1_ = *μ*_2_ and *σ*_1_ = *σ*_2_. The likelihood ratio derives as

$$\begin{array}{c} Q_{n}=\prod_{i=1}^{2}\;{\left(n/n_{i}\right)}^{n_{i}/2}\;{\left(S_{i}/S\right)}^{n_{i}/2}, \end{array}$$

with $S_{i}=\sum_{j=1}^{n_{i}}\;\left(d_{ij}^{-1}-{\hat{\mu }}_{i}^{-1}\right)$ for *i* = 1, 2, $S_{3}=n_{1}/{\hat{\mu }}_{1}+n_{2}/{\hat{\mu }}_{2}-n^{2}{\left(n_{1}{\hat{\mu }}_{1}+n_{2}{\hat{\mu }}_{2}\right)}^{-1}$, *n* = *n*_1_ + *n*_2_ and *S* = *S*_1_ + *S*_2_ + *S*_3_. Under *H*_0_ the quantity $Q_{n}^{*}\;≔\;-2\left(1-1/6\left[1/n_{1}+1/n_{2}\right]-1/\left[12n\right]\right)\log Q_{n}$ is approximately chi-square distributed with two degress of freedom. Thus, the test rejects the null hypothesis at level 5% if $Q_{n}^{*}$ exceeds the 95%-th-quantile of the *χ*^2^(2)-distribution. In the sample data set, the likelihood ratio test did not reject the null hypothesis of equal parameter sets in 83 out of 105 subjects (about 79%). For a comparison, we performed 10000 permutations by randomly assigning a first trial of one subject to a second trial of another subject and performing the likelihood ratio tests on the permuted data sets. In the mean, the null hypothesis was not rejected in only about 36% of the randomly assigned pairs, with a maximum percentage across all permutations of 51%.

In summary, the response patterns of the same subject across multiple sessions showed a high degree of reproducibility, with a Pearson correlation coefficient of up to *r* = 0.76 between log(*μ*_1_) and log(*μ*_2_) (Fig 8). The similarity of response patterns for the same subject across multiple sessions was significantly higher than the similarity of response patterns between subjects.

**Fig 9:**
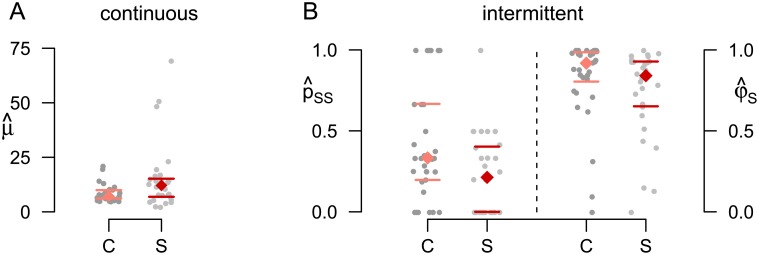
Differences in the HMM parameter estimates between subjects with schizophrenia and control subjects. (A) $\hat{\mu }$ during continuous presentation and ${\hat{p}}_{SS}$ and ${\hat{\varphi }}_{S}$ for intermittent presentation (B). Each grey dot indicates one individual participant’s data, colored diamonds indicate medians, horizontal bars indicate 25%/75%-quantiles.

*Group differences*. Finally, the HMM provides a relation between the underlying model parameters and the observed group differences reported in the introduction and in [10]. In the continuous case, the decreased alternation rate in the patients with schizophrenia is simply reflected in an increased mean dominance time $\hat{\mu }$ (Fig 9A) in the one-state HMM. For intermittent presentation, we had observed an increased alternation rate in the patients with schizophrenia. The interpretation of this observation was not obvious due to the high variability of response patterns and particularly due to the fluctuation between stable and unstable state. The HMM provides a first explanation of this phenomenon by capturing important response properties in the parameter estimates, which showed the following group differences: In particular, the expected relative time spent in the stable state,

$$\varphi_{S}\;≔\;\frac{E[\text{length}\;\text{of}\;\text{a}\;\text{stable}\;\text{phase}]}{E[\text{length}\;\text{of}\;\text{a}\;\text{stable}\;\text{phase+length}\;\text{of}\;\text{an}\;\text{unstable}\;\text{phase}]}$$

was higher in the control group (Fig 9B, detailed formula given in Eq (13) in the Materials and methods). As the main variable contributing to this difference, we observe that the probability ${\hat{p}}_{SS}$ to stay in the stable state was higher in healthy controls. In addition, the mean dominance time ${\hat{\mu }}_{U}$ in the unstable state was slightly larger in the patients with schizophrenia. The degree of statistical significance was highly similar to the one reported in [10] (*p* < .1 for $\hat{\mu }$, ${\hat{\mu }}_{U}$, ${\hat{p}}_{SS}$ and ${\hat{\varphi }}_{S}$, two-sided Wilcoxon test). In the following section, we present a model that links these observed differences to potential underlying neuronal mechanisms.

### A hierarchical Brownian motion model

As described in the previous section, the HMM captures a high variety of response patterns both in continuous and intermittent viewing, including uni- and bimodal distributions of dominance times with alternations between stable and unstable states and a high variability across subjects. With its small number of parameters, the HMM can be fitted also to short data sections available empirically and therefore also capture differences between experimental groups.

However, the HMM description remains phenomenological and does not provide insight into potential neuronal processes. Also, it cannot provide explanations for potential effects that different lengths of blank displays could have on the response patterns, as discussed for example by [14, 22, 24, 25]. In addition, the HMM cannot represent the following interesting empirical observation: Before changing from stable to unstable state, the last dominance time tends to be shorter. Therefore, we introduce here a new model, called Hierarchical Brownian Model (HBM), which provides a potential link between the phenomonological description of the response and potential underlying neuronal processes. The HBM assumptions can also provide hypotheses on the effects of different lengths of blank displays and naturally yields shorter dominance times before a state change to the unstable state.

The HBM assumes two competing neuronal populations which indicate perception of right and left rotation, respectively. As has been proposed by various authors [14, 18], we implicitly assume mechanisms of self-excitation, cross-inhibition and adaptation across these neuronal populations, without explicitly modeling them in order to reduce the number of parameters and to allow for model fitting to short trials. In order to obtain a parsimonious model description, we again assume independence of dominance times by neglecting potential mechanisms of week long-term adaptation [31]. For possible model extensions compare section ‘Applicability and model extensions’ in the discussion. We use the simplified assumption that perception arises from the difference in the activity of the two populations, which is modeled here by a Brownian motion with drift [similar to 21] that fluctuates between two thresholds, where the first passage times indicate state changes. This results in two parameters for the case of continuous presentation that are directly linked to the two parametric distribution of dominance times in the HMM. Further, we describe switching between stable and unstable states in intermittent presentation by applying an analogous mechanism, which leads to a hierarchical model. We assume another hierarchical layer of neuronal populations and a corresponding Brownian motion which modulates the drift of the first population pair and thus causes switching between stable and unstable phases.

#### Continuous presentation

The HBM in continuous presentation (HBMc) simply assumes a Brownian motion with drift ±*ν*_0_ between two borders, ±*b*, where the first hitting times of the borders indicate a percept change and lead to a sign change in the drift. As a potential neurophysiological interpretation, *b* could be considered the size of the activity difference between the *L* and *R* population required for a perception change and thereby be related to the respective population sizes. Roughly speaking, the speed of the drift *ν*_0_ could be considered related to the inverse of the connection strengths within and across populations that engage in self excitation and cross inhibition.

Formally, let *b* > 0 be a fixed border, *ν*_0_ > 0 be a drift and *T* > 0 a time horizon, and let (*W*_*t*_)_*t*∈[0,*T*]_ be a standard Brownian motion. The perception process *P* ≔ (*P*_*t*_)_*t*∈[0,*T*]_ is then defined by

$$\begin{array}{c} dP_{t}=S_{t}\nu_{0}dt+dW_{t},\;\;\text{where}\;\;P_{0}=-b, \end{array}$$

and the process *S*_*t*_ ≔ *S*(*P*_*t*_, *t*) takes the value −1 if *P*_*t*_ last hit *b* and 1 if *P*_*t*_ last hit −*b*, with *S*_0_ ≔ 1. For *t* ∈ (0, *T*] let

$$\begin{array}{c} t^{*}\;≔\;t^{*}\left(t\right)\;≔\;\sup\{x|x<t,|P_{x}\left|=b\right\},\;\text{where}\;t^{*}\left(0\right)=0 \end{array}$$

be the last time before *t* that *P*_*t*_ hit either *b* or −*b*. Then

$$\begin{array}{c} S_{t}\;≔\;S\left(P_{t},t\right)\;≔\;-\text{sgn}\left(P_{t^{*}}\right). \end{array}$$

The perception at time *t* ≥ 0 takes the value *L* (*left*) if *S*_*t*_ = 1 and *R* (*right*) if *S*_*t*_ = −1 and switches at the first-hitting times (*H*_*i*_)_*i*_ of the borders ±*b* defined by *H*_0_ ≔ 0 and

$$\begin{array}{c} H_{i}\;≔\;\inf\left\{t\leq T|t>H_{i-1},P_{t}=S_{t}b\right\}\;i=1,2,\ldots \end{array}$$ (2)

**Fig 10:**
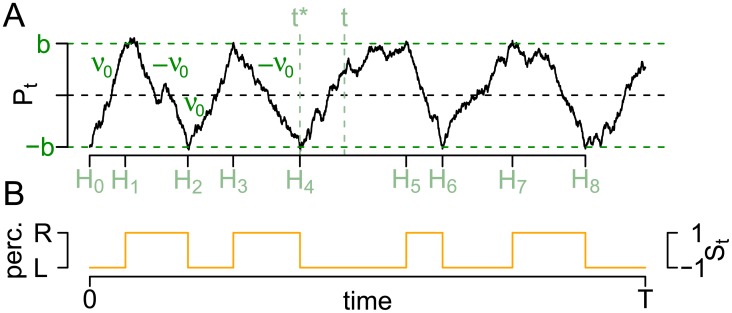
The HBMc. The first passage times (*H*_*i*_) (green) of a Brownian motion *P*_*t*_ (black, panel A) with drift ±*ν*_0_ at borders ±*b* indicate the times of the percept changes (orange, panel B). The Brownian motion is assumed to summarize the activity difference of two conflicting neuronal populations with only two parameters.

An example of such a process is shown in Fig 10. Panel A shows the process *P*, where the sign of the drift changes at each first hitting time of *b* or −*b* indicated by the process (*H*_*i*_)_*i*_, which also marks switches in the percept (panel B).

Parameter estimation in the HBMc makes use of the fact that the resulting dominance times

$$\begin{array}{c} d_{i}\;≔\;H_{i}-H_{i-1},\;\;i=1,2,\ldots \end{array}$$ (3)

are independent and IG distributed, with a known relation between the HBMc parameters *b* and *ν*_0_ and the parameters *μ* and *σ* of the IG distribution, given by *μ* = 2*b*/*ν*_0_ and $\sigma =\sqrt{2b/\nu_{0}^{3}}$ [32]. Note that the CV of the dominance time distribution is therefore given by 1/(2*bν*_0_). Thus, an increase in the border *b* also increases the mean dominance time and decreases the CV. An increase in the drift *ν*_0_ decreases the mean dominance time, while again decreasing the CV. The ML estimators $\hat{b}$ and ${\hat{\nu }}_{0}$ can be derived via

$$\begin{array}{c} \hat{b}=\left(1/2\right)\;\cdot \;\sqrt{{\hat{\mu }}^{3}/{\hat{\sigma }}^{2}}\;\text{and}\;{\hat{\nu }}_{0}=\sqrt{\hat{\mu }/{\hat{\sigma }}^{2}}, \end{array}$$

where $\hat{\mu }$ and $\hat{\sigma }$ are derived from Eq (1). Explicit expressions are given in the Materials and methods.

The precision of parameter estimates is directly comparable to the HMM for continuous presentation.

#### Intermittent presentation

**Fig 11:**
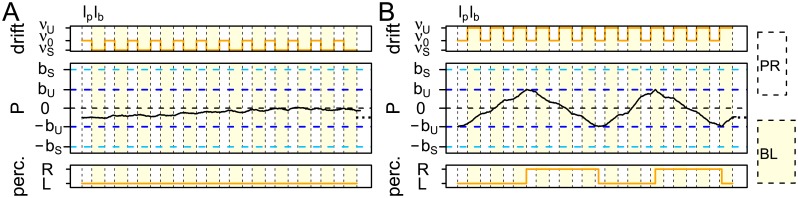
The perception process *P* in the HBMi during intermittent presentation. During presentation, *P* has drift *ν*_0_. During blank displays (yellow), *P* has drift *ν*_*S*_ in the stable phase (A), and drift *ν*_*U*_ in the unstable phase (B). Typically, we have *ν*_*S*_ ≤ *ν*_0_ and *ν*_*U*_ ≥ *ν*_0_. The borders are *b*_*S*_ (light blue horizontal line) in the stable phase and *b*_*U*_ (blue horizontal line) in the unstable phase.

In the Hierarchical Brownian Motion model for intermittent presentation (HBMi), we require mechanisms for long dominance times in the stable state as well as for short dominance times in the unstable state. In order to describe the responses to intermittent and continuous presentation in one model framework, we assume the identical perceptual process as in the HBMc during phases of stimulus presentation. The periods of blank display represent the only difference to continuous presentation. In these periods, we assume additional neuronal mechanisms. In particular, we assume that the perceptual process then takes on one of two mean drifts, *ν*_*S*_ in the stable state and *ν*_*U*_ ≥ *ν*_*S*_ in the unstable state, with potentially opposite signs of *ν*_0_ and *ν*_*S*_ for increased stability (Fig 11). Note that the drifts *ν*_*S*_ and *ν*_*U*_ are not necessarily constant across the whole period of blank display, but they denote the mean drift of the process, which is sufficient to describe the distribution of dominance times. Interestingly, additional assumptions on the temporal behavior of the drift terms could also allow describing the impact of the lengths of blank displays (cmp. Discussion). Further, in the unstable state the border *b*_*U*_ at which perception and drift direction change is assumed smaller than the border *b*_*S*_ during stable perception. Switches between the stable and unstable state will be caused by a similar mechanism in a so-called background process *B* described later in this section.

Within a state (*S* or *U*), the fluctuation of the perception process between the borders is assumed analogous to the HBMc, except that the borders are dependent on the hidden state and that the drift is *ν*_0_ during presentation and *ν*_*S*_ or *ν*_*U*_ during blank display. Formally, we denote by *PR* and *BL* the sets of all periods of stimulus presentation and blank display, respectively. Assuming that we start a trial with a presentation interval and then switch regularly between presentation intervals of length *l*_*p*_ and blank display of length *l*_*b*_, *PR* and *BL* are given by

$$\begin{array}{ccc} \text{PR} & = & \overset{T/l_{b}}{\underset{i=1}{⋃}}\;\left[\left(i-1\right)\left(l_{p}+l_{b}\right);\left(i-1\right)\left(l_{p}+l_{b}\right)+l_{p}\right)\\ \text{BL} & = & \overset{T/l_{b}}{\underset{i=1}{⋃}}\;\left[\left(i-1\right)\left(l_{p}+l_{b}\right)+l_{p};i\left(l_{p}+l_{b}\right)\right)\;(\text{Fig}\;11). \end{array}$$

The perception process (*P*_*t*_)_*t*_ is then given by

$$\begin{array}{c} dP_{t}=\{\begin{array}{cc} S_{t}\nu_{0}dt+dW_{t}, & \;\text{if}\;t\in \text{PR}\\ S_{t}\nu_{{\tilde{Y}}_{t}}dt+dW_{t}, & \;\text{if}\;t\in \text{BL}, \end{array} \end{array}$$

where ${\tilde{Y}}_{t}\in \left\{S,U\right\}$ denotes the hidden state and (*W*_*t*_)_*t*_ denotes a standard Brownian motion. As a result, the mean drift per second is given by

$$\begin{array}{c} \nu_{S}^{*}\;≔\;\frac{l_{b}\;\cdot \;\nu_{S}+l_{p}\;\cdot \;\nu_{0}}{l_{b}+l_{p}}\;\;\text{and}\;\;\nu_{U}^{*}\;≔\;\frac{l_{b}\;\cdot \;\nu_{U}+l_{p}\;\cdot \;\nu_{0}}{l_{b}+l_{p}} \end{array}$$ (4)

for states *S* and *U*, respectively. Because the periods *l*_*b*_ and *l*_*p*_ are typically short in relation to a dominance time, the behavior of *P* can be approximated by a Brownian motion with absolute drifts $\nu_{S}^{*}$ and $\nu_{U}^{*}$, respectively. As in the HBMc, the sign of the drift *S*_*t*_ ≔ *S*(*P*_*t*_, *t*) changes at every first hitting time of the respective border, i.e.,

$$\begin{array}{c} S\left(P_{t},t\right)\;≔\;-\text{sgn}\left(P_{t^{*}}\right)\;\text{where}\;t^{*}\;≔\;t^{*}\left(t\right)\;≔\;\sup\{x|x<t,|P_{x}\left|=b_{{\tilde{Y}}_{x}}\right\}\;\text{with}\;t^{*}\left(0\right)=0. \end{array}$$

We initialize $P_{0}=-b_{{\tilde{Y}}_{0}}$ for the initial state ${\tilde{Y}}_{0}$ which is the stable state with probability $\pi_{S}\;≔\;P\left({\tilde{Y}}_{0}=S\right)$. The perception then takes the value *L* if *S*_*t*_ = 1 and *R* if *S*_*t*_ = −1 and switches at the first-hitting times (*H*_*i*_)_*i*_ of the borders ±*b*_*i*_ comparable to Eq (2). Note that perception also changes during blank display. The dominance times are therefore again given by *d*_*i*_ ≔ *H*_*i*_ − *H*_*i*−1_, *i* = 1, 2, ….

**Fig 12:**
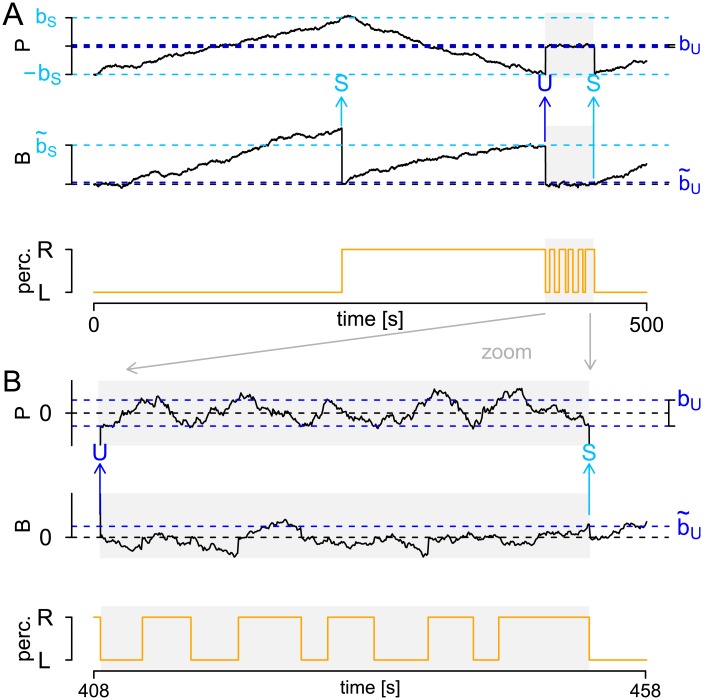
The HBMi. The perception process *P*, the background process *B* and the resulting percept. A: A simulation on [0, 500]. B: The same realization, zoomed in on the time interval [408, 458]. Stable phases indicated by white background, unstable phases indicated by gray background. The beginnings of stable and unstable phases are marked with light blue and blue arrows, respectively.

In order to describe the switching between the two states *S* and *U*, we use an analogous upper hierarchical level with another pair of conflicting neuronal populations. Their difference activity is described by a so-called background process *B* ≔ (*B*_*t*_)_*t*_ (Fig 12A, middle panel). *B* is also assumed to be a Brownian motion with drift. Its drift is assumed to vanish during presentation and to take the value *ν*_*B*_ > 0 during blank display, i.e.,

$$\begin{array}{c} dB_{t}=\{\begin{array}{cc} d{\tilde{W}}_{t}, & \;\text{if}\;t\in \text{PR}\\ \nu_{b}dt+d{\tilde{W}}_{t}, & \;\text{if}\;t\in \text{BL},{\tilde{Y}}_{t}=S\\ -\nu_{b}dt+d{\tilde{W}}_{t}, & \;\text{if}\;t\in \text{BL},{\tilde{Y}}_{t}=U, \end{array} \end{array}$$ (5)

where ${\left({\tilde{W}}_{t}\right)}_{t}$ is a Brownian motion independent of (*W*_*t*_)_*t*_. Again, the mean drift across PR and BL intervals is $\nu_{B}^{*}\;≔\;\frac{l_{b}\cdot \nu_{B}}{l_{b}+l_{p}}$.

The background process *B* evokes changes between the stable and the unstable state. Specifically, at the time of a percept change t*, the question of whether the process stays in the former state (*S* or *U*) or switches to the other state depends only on the value of *B*. Two borders, *b*_*U*_ < *b*_*S*_ determine this switching as follows (see Fig 12). If the former state is *S*, the process remains stable if and only if $B_{t^{*}}>{\tilde{b}}_{S}$ (first light blue arrow in panel A), while switching to the unstable state if $B_{t^{*}}\leq {\tilde{b}}_{S}$ (blue arrow, panel A). Analogously, if the former state is *U*, the process switches to *S* if and only if $B_{t^{*}}>{\tilde{b}}_{U}$ (right light blue arrow, panel A), while staying in *U* if $B_{t^{*}}\leq {\tilde{b}}_{U}$ (blue arrow, panel B). After the percept change, the background process *B* is reset to zero and then follows its usual dynamic (Eq (5)), i.e., the sign of its drift changes if and only if the state has changed. Finally, as the perception process *P* fluctuates between ±*b*_*S*_ in the stable state and between ±*b*_*U*_ in the unstable state, the value of *P* is reset when the state changes, to the value sgn(*P*)*b*_*S*_ when changing to the stable state and to sgn(*P*)*b*_*U*_ when changing to the unstable state.

#### Discussion of assumptions and interpretation of parameters

**Fig 13:**
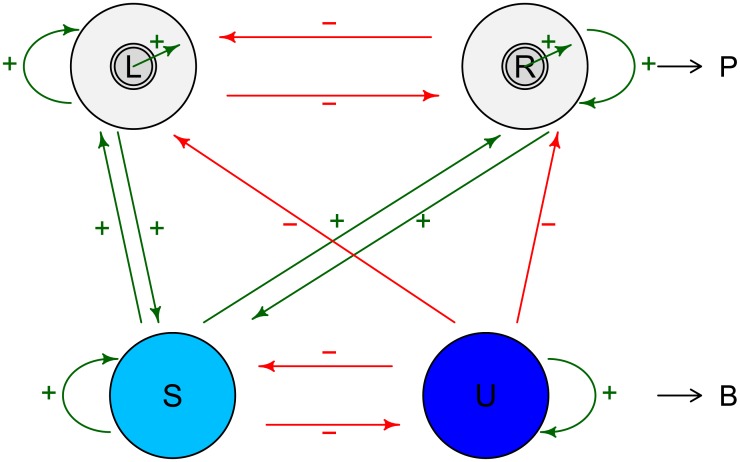
Motivation of HBMi model assumptions. For explanation and details see text.

The technical advantage of the HBMi is that the resulting dominance times agree in most parts with the dominance times resulting from the HMM assumptions, which allows model fitting also to short data sections and comparison across clinical groups. In addition, the HBMi also provides a relation to potential underlying neuronal processes, as discussed in the following and illustrated in Fig 13.

Both HBMi-processes *P* and *B* are assumed Brownian motions with drift which may be interpreted as the activity difference between neuronal populations. Implicitly, this assumes mechanisms of self-excitation, cross-inhibition and adaptation across these neuronal populations, as proposed by various authors [14, 18]. Without explicitly modeling such mechanisms in order to reduce the number of parameters and allow model fitting, the parameter sets are reduced to the mean drifts *ν* and the borders *b*. Analogously to the HBMc, the speed of the drifts could be considered related to the inverse of the connection strengths within and across populations that engage in self excitation and cross inhibition. The border *b*, in analogy to the HBMc, could be considered related to the size of the respective populations under consideration. The use of different borders allows fitting of highly various response patterns and can be motivated as follows.

In the HBMi, the perception process *P* has two borders, *b*_*S*_ > *b*_*U*_ for the stable and the unstable state. This suggests different population sizes of neurons involved in the stable and unstable state. Typically *b*_*S*_ > *b* > *b*_*U*_, suggesting that in the stable state, the activity of the dominant population is increased by joining additional neurons to the population, for example by positive feedback mediated by population *S*. Vice versa, in the unstable state, only a minimal population is involved in the respective percept, leading to fast changes. Thus, one could assume that the dominant percept population size is decreased by the population *U* (red arrows). The active population sizes are indicated by different circle sizes in the first line of Fig 13 and are assumed modulated by the background populations *S* and *U*.

The background process *B* models the activity difference between *S* and *U* and is also associated with two borders, ${\tilde{b}}_{S}$ and ${\tilde{b}}_{U}$. The assumption regarding resetting of *B* at percept change is technically necessary to generate independent dominance times and to thus allow straightforward model fitting (cmp. section ‘Parameter estimation’). In the picture of Fig 13 it can be motivated as follows. Population *S* is capable of offering positive feedback to the currently active population, *L* or *R*, which results in an increased population size as described above. *S* is also activated by the active population. Therefore, a percept change causes a reseting to zero. However, if *S* had shown high previous activation (above ${\tilde{b}}_{S}$), the activity of *S* can increase rapidly again, causing another stable dominance time. In contrast, in case of weak previous activation (below ${\tilde{b}}_{S}$), the unstable population *U* is taking over, marking the transition to an unstable state. With opposite signs, i.e., negative drift and a small new border ${\tilde{b}}_{U}$, the process proceeds analogously. Similar to the mean drift terms *ν*_*S*_ and *ν*_*U*_, the drift *ν*_*B*_ is not necessarily constant but describes the mean drift of *B* during the period of blank display.

In addition to the potential neurophysiological interpretations of the model parameters, we give here a relation of the parameters to the response patterns. Interestingly, the seven HBMi parameters allow the reproduction of highly variable response patterns as are also observed in the empirical data sets (e.g., Fig 1). The following quantities, which are easily derived from the parameters, offer a straightforward pattern interpretation.

First, the parameter sets $\left(b_{S},\nu_{S}^{*}\right)$ and $\left(b_{U},\nu_{U}^{*}\right)$ can be interpreted analogously to the parameters (*b*, *ν*_0_) in continuous stimulation. That means, an increase in the border (*b*_*S*_ or *b*_*U*_) increases the mean dominance time and decreases the CV in the respective state. An increase in the drift ($\nu_{S}^{*}$ or $\nu_{U}^{*}$) decreases the mean dominance time, while also decreasing the CV. Recall that the CVs of dominance times during stable and unstable states are given by

$$\begin{array}{c} {\text{CV}}_{S}^{*}\;≔\;1/\sqrt{2b_{S}\nu_{S}^{*}}\;\;\text{and}\;\;{\text{CV}}_{U}^{*}\;≔\;1/\sqrt{2b_{U}\nu_{U}^{*}},\;\text{respectively}. \end{array}$$

**Fig 14:**
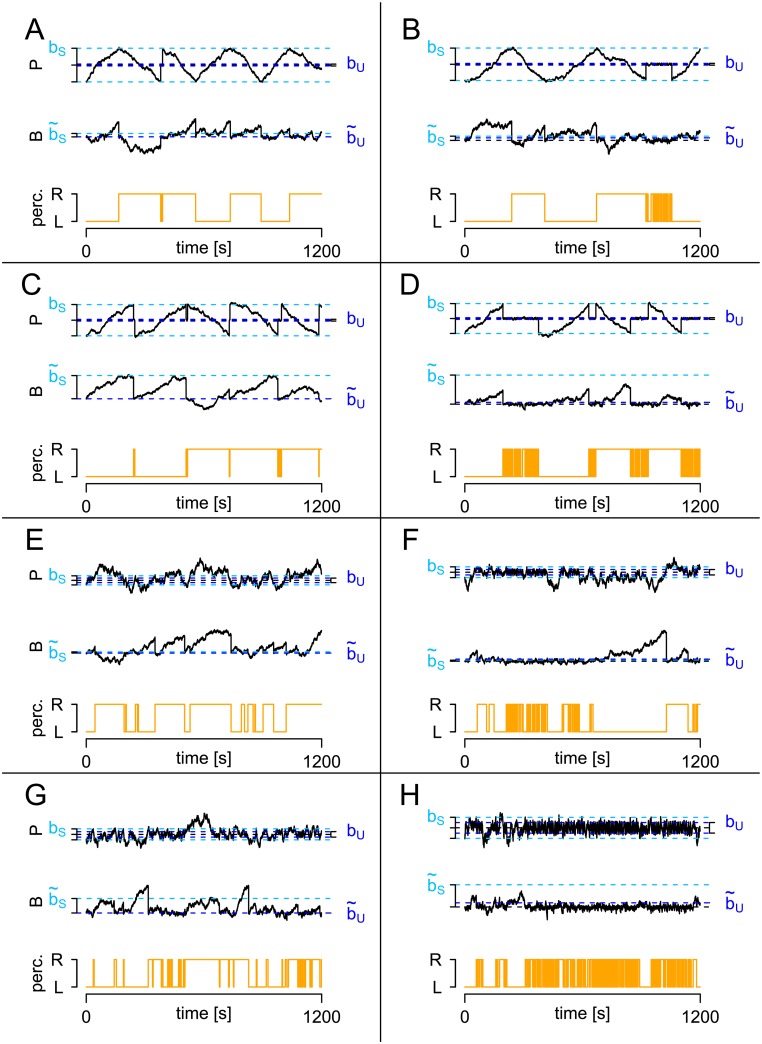
Impact of HBMi parameter values on the response patterns. Examples of simulated response patterns are shown for different values of the three quantities ${\text{CV}}_{S}^{*}$, $2b_{S}\nu_{B}^{*}/{\tilde{b}}_{S}\nu_{S}^{*}$ and ${\tilde{b}}_{U}$. The quantities for panels A-H were ${\text{CV}}_{S}^{*}=\left\{0.2,0.2,0.2,0.2,1,1,1,1\right\}$, $2b_{S}\nu_{B}^{*}/{\tilde{b}}_{S}\nu_{S}^{*}=\left\{4,4,0.4,0.4,4,4,0.4,0.4\right\}$, ${\tilde{b}}_{U}=\left\{0,3,0,3,0,3,0,3\right\}.$

Fig 14 illustrates examples with small ${\text{CV}}_{S}^{*}$ (panels A-D) and large ${\text{CV}}_{S}^{*}$ (panels E-H).

Second, the parameters ${\tilde{b}}_{S}$ and $\nu_{B}^{*}$ can be interpreted best when compared to *b*_*S*_ and $\nu_{S}^{*}$ as follows. Consider the expected fraction of ${\tilde{b}}_{S}$ reached by the background process at the end of a stable dominance time,

$$\begin{array}{c} \frac{\text{Expected}\;\text{duration}\;\text{of}\;\text{a}\;\text{stable}\;\text{dominance}\;\text{time}}{\text{Expected}\;\text{duration}\;\text{until}\;B\;\text{reaches}\;{\tilde{b}}_{S}}=\frac{2b_{S}/\nu_{S}^{*}}{{\tilde{b}}_{S}/\nu_{B}^{*}}=\frac{2b_{S}\nu_{B}^{*}}{{\tilde{b}}_{S}\nu_{S}^{*}}, \end{array}$$

which is related to the transition probability from stable to unstable state. In case of a small background border ${\tilde{b}}_{S}<b_{S}$ and small $\nu_{S}^{*}$, the probability of *B* crossing ${\tilde{b}}_{S}$ until percept change is high, such that the process remains stable. Fig 14A, 14B, 14E and 14F show such parameter combinations. An analogous term can be derived in comparison to the parameters ${\tilde{b}}_{U}$ and $\nu_{U}^{*}$.

Third, the parameter ${\tilde{b}}_{U}$ is related to the number of dominance times in the unstable state before changing to the stable state. Recall that the drift of *B* is negative during unstable periods. Therefore, a large value of ${\tilde{b}}_{U}$ implies a low probability to reach ${\tilde{b}}_{U}$ until the percept change. This implies a high expected number of dominance times in the unstable state, or a low transition probability from the unstable to the stable state. Fig 14B, 14D, 14F and 14H show examples with large values of ${\tilde{b}}_{U}$.

#### Relation of the HBMi to the two state HMM

The relation of the HBMc to the one state HMM is simple as is represents only a reparameterization. Both the one state HMM and the HBMc yield independent and IG distributed dominance times. For the intermittent case, the relation of the HBMi to the two state HMM is not as straightforward. The two models are highly similar in the sense that they use two parameters to describe long and short dominance times, respectively (e.g., (*μ*_*S*_, *σ*_*S*_) and $\left(b_{S},\nu_{S}^{*}\right)$ for the stable state). In the HMM, the dominance times are IG distributed, given the state with the respective parameters. In the HBMi, the dominance times are approximately IG distributed, where the minor deviation from the IG distribution originates from the minor deviation of *P* from a Brownian motion with drift $\nu_{S}^{*}$ (or $\nu_{U}^{*}$), instead of exactly assuming drift *ν*_0_ during stimulation and *ν*_*S*_ (or *ν*_*U*_) during blank display. However, the marginal distribution of *P* at multiples of such intervals *l*_*p*_ + *l*_*b*_ is identical to the marginal distribution of a Brownian motion with drift $\nu_{S}^{*}$ (or $\nu_{U}^{*}$) at these time points, and the differences can only be observed in the meantime. Because dominance times usually span multiple trials of duration *l*_*p*_ + *l*_*b*_, the approximation is very close. As another similarity, both models use additional parameters ((*p*_*SS*_, *p*_*UU*_) and $\left({\tilde{b}}_{S},{\tilde{b}}_{U},\nu_{B}\right)$) to describe the transition probabilities between the stable and the unstable state.

The main difference between the HBMi and the two state HMM concerns the dynamic of the state transitions between stable and unstable state. In the HMM, transition probabilities are given by (1 − *p*_*SS*_) and (1 − *p*_*UU*_) and are independent of the duration of the previous dominance time. In contrast, in the HBMi, a transition from stable to unstable state requires that *B* has not reached ${\tilde{b}}_{S}$ at the end of the respective dominance time. Therefore, the transition probability ${\tilde{p}}_{SU}\left(d_{i}\right)$ depends on the duration *d*_*i*_ of the *i*-th dominance time, where shorter dominance times yield higher transition probabilities. Note that the position of *B* at the end of a dominance time *d*_*i*_ is given by an increment of a Brownian motion with drift $\nu_{B}^{*}$ in the fixed time interval *d*_*i*_. Therefore the position is normally distributed with mean $d_{i}\;\cdot \;\nu_{B}^{*}$ and variance *d*_*i*_ and the probability to remain in the stable state (which is the probability that the background process exceeds ${\tilde{b}}_{S}$) is given by

$${\tilde{p}}_{SS}\left(d_{i}\right)=P\left(Y_{i+1}=S|Y_{i}=S,d_{i}\right)\approx 1-\Phi_{\nu_{B}^{*}d_{i},d_{i}}({\tilde{b}}_{S}),$$ (6)

where Φ_*μ*,*σ*^2^_() denotes the distribution function of the normal distribution with mean *μ* and variance *σ*^2^ and *Y*_*i*_ is the hidden state of the *i*-th dominance time. Analogously, for the transition from unstable to stable state, the transition probability ${\tilde{p}}_{US}\left(d_{i}\right)$ tends to decrease with longer dominance times, and we find

$${\tilde{p}}_{UU}\left(d_{i}\right)=P\left(Y_{i+1}=U|Y_{i}=U,d_{i}\right)\approx \Phi_{-\nu_{B}^{*}d_{i},d_{i}}({\tilde{b}}_{U}).$$ (7)

Note that we use the approximate sign ‘≈’ because the drift of *B* is not exactly $\nu_{B}^{*}$ throughout, but is assumed to change between *ν*_0_ and *ν*_*B*_ during stimulation and blank display, respectively, yielding a mean drift of $\nu_{B}^{*}$. Analogously to the above explanation, differences caused by the approximation can be considered minimal.

In order to obtain quantities comparable to the transition probabilities *p*_*SS*_ and *p*_*UU*_ in the HMM, we derive the marginal transition probabilities in the HBMi as the expected value of ${\tilde{p}}_{SS}$ and ${\tilde{p}}_{UU}$. As shown by [39], the positions *X*_*S*_ and *X*_*U*_ of *B* at the end of an independent stable or unstable IG distributed dominance time follow the Normal Inverse Gauss (NIG) distribution. The resulting transition probabilities in the HBMi can then be calculated as

$$\begin{array}{c} p_{SS}^{*}=P\left(X_{S}>{\tilde{b}}_{S}\right)\;\;\text{and}\;\;p_{UU}^{*}=P\left(X_{U}\leq {\tilde{b}}_{U}\right). \end{array}$$ (8)

where *X*_*S*_ is NIG-distributed with parameters (0, $\sqrt{{\nu_{S}^{*}}^{2}+{\nu_{B}^{*}}^{2}}$, $\nu_{B}^{*}$, 2*b_S_*) and *X*_*U*_ is NIG-distributed with parameters (0, $\sqrt{{\nu_{U}^{*}}^{2}+{\nu_{B}^{*}}^{2}}$, $-\nu_{B}^{*}$, 2*b_U_*).

One should note that due to the difference in transition probabilities of the two models, the parameters $\left(b_{S},\nu_{S}^{*}\right)$ are not direct reparameterizations of (*μ*_*S*_, *σ*_*S*_) (and similarly for the unstable state). Furthermore, the dependence of the transition probability on the length of the previous dominance time is one important new aspect of the HBMi not described in the HMM, which will also be used in the section ‘Application of the HBM to the sample data set’ for comparison of models and empirical observations.

#### Parameter estimation

In order to estimate the HBMi parameter set $\Theta =(b_{S},\nu_{S}^{*},b_{U},\nu_{U}^{*},{\tilde{b}}_{S},{\tilde{b}}_{U},\nu_{B}^{*})$, we use ML estimation. The likelihood *L* is given by

$$\begin{array}{c} L\left(d_{1},\ldots ,d_{n}|\Theta \right)\approx \alpha_{S}\left(n\right)+\alpha_{U}\left(n\right), \end{array}$$ (9)

where the forward variable *α*_*j*_(*i*) denotes the probability of being in state *j* at time *i* while observing (*d*_1_, …, *d*_*i*_). The approximation is again due to the averaging of drifts during blank display and stimulus presentation. The forward variables can be derived recursively [34, 40] by

$$\begin{array}{c} \alpha_{j}\left(i\right)=\{\begin{array}{cc} \pi_{j}f_{j}\left(d_{i}\right) & \;\text{if}\;i=1\\ f_{j}\left(d_{i}\right)\;\sum_{j\in \{S,U\}}\;\alpha_{j}\left(i-1\right){\tilde{p}}_{ij}\left(d_{i-1}\right) & \;\text{if}\;i>1 \end{array} \end{array}$$ (10)

with *π*_*j*_ as starting distribution and *f*_*S*_ and *f*_*U*_ denoting the densities of $\text{IG}(2b_{S}/\nu_{S}^{*},\sqrt{2b_{S}/{\nu_{S}^{*}}^{3}})$ and $\text{IG}(2b_{U}/\nu_{U}^{*},\sqrt{2b_{U}/{\nu_{U}^{*}}^{3}})$ distributed random variables, respectively. Details on the maximization algorithm can be found in the Materials and methods. After estimation of Θ, the estimates of ${\hat{\nu }}_{S}$ and ${\hat{\nu }}_{U}$ can be obtained using Eq (4), and analogously for ${\hat{\nu }}_{B}$.

**Fig 15:**
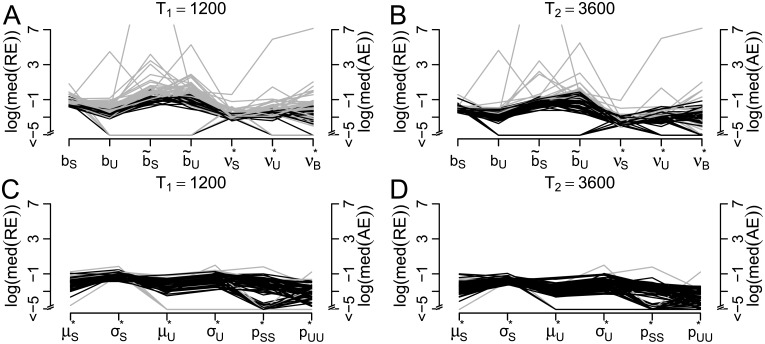
Precision of parameter estimates in the HBMi. For each of the 61 parameter constellations estimated from the sample data set, 1000 simulations were performed with the HBMi. (A) and (B): log(median(REs)) of the border parameters *b_S_*, *b_U_*, ${\tilde{b}}_{S}$, ${\tilde{b}}_{U}$, and log(median(AEs)) of the drift parameters $\nu_{S}^{*}$, $\nu_{U}^{*}$, $\nu_{B}^{*}$ with *T* = 1200*s* (A) and *T* = 3600*s* (B). (C) and (D) log(median(REs)) for the derived model parameters $\mu_{S}^{*}$, $\sigma_{S}^{*}$, $\mu_{U}^{*}$, $\sigma_{U}^{*}$ and log(median(AEs)) for the parameters $p_{SS}^{*}$ and $p_{UU}^{*}$ for *T* = 1200*s* (C) and *T* = 3600*s* (D). Parameter combinations with mean errors < 0.25 across all variables plotted in black.

*Precision of parameter estimation*. The variability of the parameter estimates in the HBMi is studied analogously to the HMM, using the RE and the AE of the parameter estimates obtained in 1000 simulations of the 61 parameter combinations estimated from the empirical data set. For the border parameters *b_S_*, *b_U_*, ${\tilde{b}}_{S}$, ${\tilde{b}}_{U}$, we use the RE, while for the typically small drift parameters $\nu_{S}^{*}$, $\nu_{U}^{*}$, $\nu_{B}^{*}$, the AE is used. Again, one set of simulations uses the time horizon of the empirical data, *T*_1_ = 1200s (Fig 15A), and a second simulation was performed using *T*_2_ = 3600s (Fig 15B). According to the simulation results, the precision of parameter estimation in the given parameter range is not always satisfactory for the given parameterization $\left(b_{S},b_{U},{\tilde{b}}_{S},{\tilde{b}}_{U},\nu_{S}^{*},\nu_{U}^{*},\nu_{B}^{*}\right)$, yielding average errors (i.e., mean REs or AEs across all variables) smaller than 0.25 in only 24 out of 61 cases for the empirical time horizon *T*_1_ and still in only 44 out of 61 for the tripled sample size of *T*_2_. This suggests that these raw parameters yield less reliable estimates because different combinations of *b* and *ν* can yield the same mean stable dominance time. In contrast, the following set of derived parameters that are more easily comparable to the HMM parameters shows better properties. We denote by $\left(\mu_{S}^{*},\sigma_{S}^{*}\right)$ and $\left(\mu_{U}^{*},\sigma_{U}^{*}\right)$ the mean and standard deviation of dominance times in the HBMi in the stable and unstable state, respectively. These have analogous interpretations as (*μ*_*S*_, *σ*_*S*_) and (*μ*_*U*_, *σ*_*U*_) in the HMM and are given by

$$\begin{array}{c} \mu_{S}^{*}\approx 2b_{S}/\nu_{S}^{*}\;\;\text{and}\;\;\sigma_{S}^{*}\approx \sqrt{2b_{S}/{\nu_{S}^{*}}^{3}}, \end{array}$$ (11)

and analogously for $\mu_{U}^{*}$ and $\sigma_{U}^{*}$, where the approximation is again due to the minimal difference between the mean drift $\nu_{S}^{*}$ and the changing drift *ν*_*S*_ + *ν*_0_ during presentation and blank display. In addition, we consider the transition probabilities between the states, $p_{SS}^{*}$ and $p_{UU}^{*}$ (cmp. Eq (8)). Fig 15C and 15D show the median REs for $\mu_{S}^{*}$, $\sigma_{S}^{*}$, $\mu_{U}^{*}$, $\sigma_{U}^{*}$ and median AEs for $p_{SS}^{*}$, $p_{UU}^{*}$ obtained in the 1000 simulations of length *T*_1_ (panel C) and *T*_2_ (panel D). Concerning this parameterization, 51 parameter combinations yielded average errors smaller than 0.25 across all variables for *T*_1_, while as many as 58 out of 61 cases showed errors smaller than 0.25 for *T*_2_. These simulations suggest that the parameterization $\left(\mu_{S}^{*},\sigma_{S}^{*},\mu_{U}^{*},\sigma_{U}^{*},p_{SS}^{*},p_{UU}^{*}\right)$ yields more reliable parameter estimates in the HBMi than the original parameterization of borders and drifts.

#### Application of the HBM to the sample data set

For the analysis of the sample data set, the HBMc and HBMi were fitted to the dataset of [10] containing responses to continuous and intermittent stimulation for each of 29 patients with schizophrenia and 32 control subjects, using the parameter estimation described in the section ‘Parameter estimation’.

**Fig 16:**
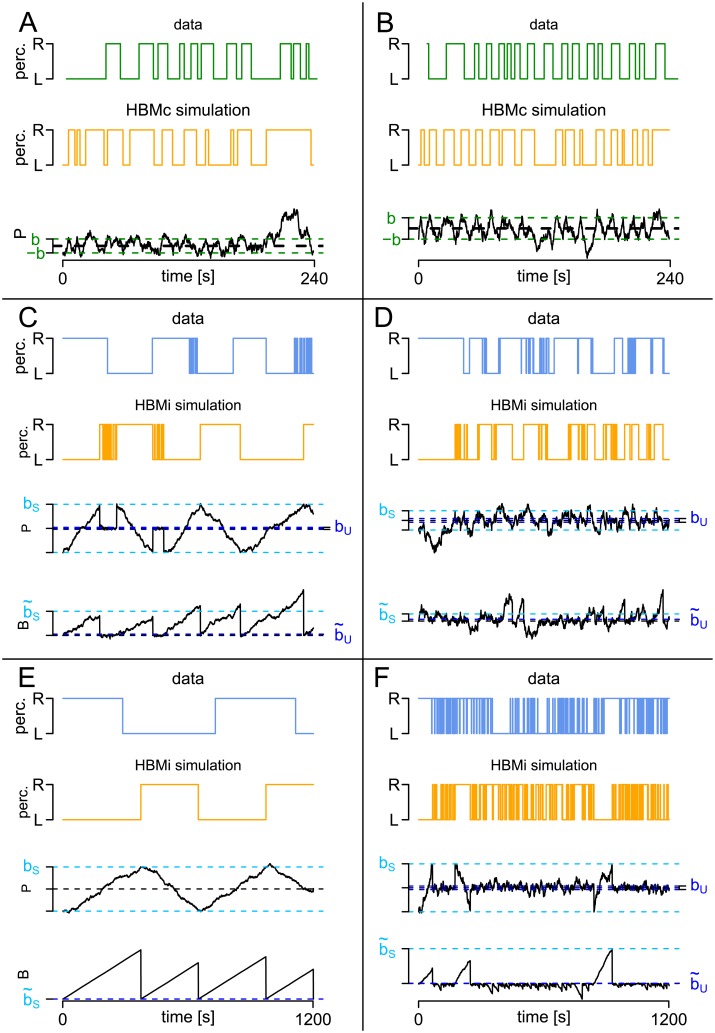
HBM: Examples of simulated response patterns. Example empirical response patterns from Fig 1 and response patterns simulated by the HBM (orange). Responses to continuous and intermittent presentation are plotted in green and blue, respectively. The estimated parameters used for simulation are given in Tables 3 and 4, respectively. (A) and (B) Continuous presentation and HBMc. (C)-(F): Intermittent presentation and HBMi. Below the orange response patterns, one can see the perception process *P* and (for the HBMi) the background process *B* corresponding to the respective simulation.

*Model fit*. Because the HBMc represents only a reparameterization of the one-state HMM, results are completely analogous for continuous presentation. Thus, a high variability of response patterns can be described with the two parametric distribution (see Fig 16A and 16B), including also different means and variances of dominance times. For intermittent presentation, the HBMi and the two-state HMM are similar, but also show a number of differences. As a first similarity to the two-state HMM, the HBMi can also describe and reproduce a high variety of response pattens (Fig 16C–16F). For example, these include highly regular stable states that may or may not be interrupted by short unstable phases (C,E) or response patterns with different degrees of regularity (D, F) and different alternation rates. Note also that the response patterns to intermittent stimulation of six out of the 61 subjects were described better by the one-parametric HBMc (e.g., E).

**Table 3:** Estimated HBMi parameter combinations of the typical response pattens to continuous presentation shown in Fig 1A and 1B.

com.	$\hat{b}$	${\hat{\nu }}_{0}$
A	2.08	0.40
B	2.42	0.72

**Table 4:** Estimated HBMi parameter combinations of the typical response pattens to intermittent presentation shown in Fig 1C–1F.

com.	${\hat{\nu }}_{0}$	${\hat{b}}_{S}$	${\hat{\nu }}_{S}$	${\hat{b}}_{U}$	${\hat{\nu }}_{U}$	${\hat{\tilde{b}}}_{S}$	${\hat{\tilde{b}}}_{U}$	${\hat{\nu }}_{B}$
C	0.72	41.74	0.24	1.84	0.74	49.71	2.39	0.54
D	0.27	5.42	0.08	1.06	0.93	4.56	1.13	0.06
E	0.32	52.58	0.25	NA	NA	0.00	NA	17.5
F	0.57	13.5	0.24	1.00	0.24	110.28	0.77	1.90

**Fig 17:**
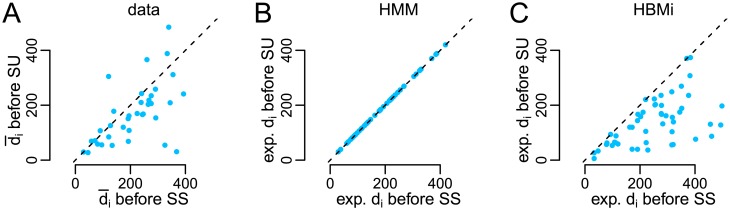
Mean dominance times in stable state as a function of the successive state. In the empirical data set, the mean dominance time before a state transition *SU* is shorter than the mean dominance time before *SS* (A). This observation can be reproduced in the HBMi (C), while the expected dominance time in the HMM (B) is equally long for intervals with and without transition, i.e., independent from transition between states. In the empirical data set [10], the state transitions were estimated using the Viterbi paths [34]. Analogous results were obtained using a fixed threshold. For the HBMi, the expected dominance times were derived from the HBMi parameters according to Eq (15) in the Materials and methods. In the HMM, expected dominance times correspond to ${\hat{\mu }}_{S}$.

In addition to the close description and reproduction of the patterns in the empirical data, one interesting additional aspect is captured by the HBMi, which cannot be described in the two-state HMM. As explained in the section ‘Relation of the HBMi to the two state HMM’, the probability of a transition from stable to unstable state decreases with the length of the dominance time in the HBMi. The same is true for the reverse transition. Indeed, the same observation can be made in the empirical data set, while this dependence cannot be captured within the HMM (Fig 17).

*Group differences*. Due to the high correspondence of the HBMi response patterns with the empirical data and the neurophysiologically related parameterization, the HBMi provides potential additional links to underlying neuronal processes of the observed differences between control subjects and patients with schizophrenia. Here, we consider three aspects related to continuous and intermittent stimulation and to the transition between these two conditions.

**Fig 18:**
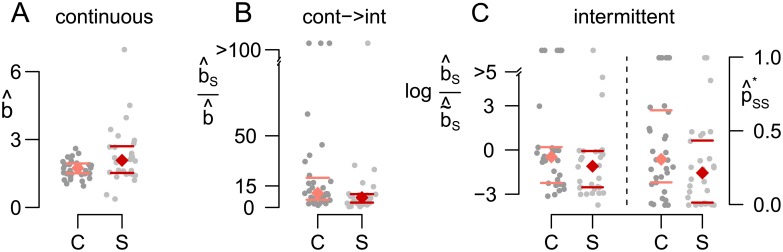
Group differences between patients with schizophrenia and control subjects in the HBM. The HBMc parameter $\hat{b}$ (A) and the relation $\hat{b}/{\hat{b}}_{S}$ (B). (C): Differences in ${\hat{b}}_{S}/{\hat{\tilde{b}}}_{S}$ and ${\hat{p}}_{SS}^{*}$. The raw data together with the median (colored diamonds) and 25%- and 75%-quantiles are shown. All *p*-values of a two-sided Wilcoxon test were below.1.

First, concerning continuous stimulation, we note that the one-state-HMM and the HBMc yield identically distributed sequences of dominance times. Note that the mean dominance time $\hat{\mu }$ in the HMM (Fig 9A) therefore equals the corresponding value $2\hat{b}/{\hat{\nu }}_{0}$ in the HBMc. As a consequence, the results and interpretation were identical, i.e., the higher alternation rate of the control subjects during continuous presentation (Fig 2) was reflected in a smaller value of $2\hat{b}/{\hat{\nu }}_{0}$. When analyzing the individual parameters *b* and *ν*_0_, we found no group differences in the drift *ν*_0_, but a tendency for a larger neuronal pool *b* involved in sensory processing in the group of patients with schizophrenia (Fig 18A, *p* < .1, two-sided Wilcoxon test).

Second, regarding intermittent presentation, we focussed on the derived parameters $\left(\mu_{S}^{*},\sigma_{S}^{*},\mu_{U}^{*},\sigma_{U}^{*},p_{SS}^{*},p_{UU}^{*}\right)$, which show correspondence to the HMM and good estimation properties, instead of testing the border and drift parameters individually. Similar to the HMM, we found an increased mean unstable dominance time in the group of patients with schizophrenia as compared to healthy controls. More importantly and also consistent with the HMM, we also found that patients with schizophrenia showed a decreased relative time spent in *S*, $\varphi_{S}^{*}$ (*p* < .1, two-sided Wilcoxon test, see Eq (17) in the Materials and methods). Again, the probability $p_{SS}^{*}$ to stay in the stable state seemed to be the main variable contributing to this difference, being significantly reduced in patients with schizophrenia (Fig 18C, *p* < .1, two-sided Wilcoxon test). In order to identify potential neurophysiological mechanisms underlying this group difference, we further investigated the components of $p_{SS}^{*}$. Noting that no difference was observed in the mean stable dominance time $\mu_{S}^{*}=2b_{S}/\nu_{S}^{*}$ and in the relation $\nu_{S}^{*}/\nu_{B}^{*}$ of the drift in stable state and the drift of the background process, one interesting parameter is $b_{S}/{\tilde{b}}_{S}$. Keeping all other parameters constant, an increase in this quantity means an increase in $p_{SS}^{*}$ because the crossing of ${\tilde{b}}_{S}$ at the end of a stable dominance time, i.e., staying in the stable state, gets more likely. In the empirical data set, we found increased values of $b_{S}/{\tilde{b}}_{S}$ in the control group (Fig 18C, *p* < .1, two-sided Wilcoxon test). In terms of the potential neurophysiological interpretation, this would suggest an increased population size involved in stable perception processing as a potential main underlying mechanism.

Third, a similar observation resulted from the derived parameter *b*_*S*_/*b*, which describes a transition of population sizes from continuous to intermittent stimulus processing. In the data set, we observed increased values of *b*_*S*_/*b* for the control group (Fig 18B) as compared to the group of patients with schizophrenia. Again, applying a potential neurophysiological interpretation, this would suggest that in the control group, the neuronal pool that is additionally recruited during intermittent presentation in stable states could be higher than in the group of patients with schizophrenia. Note that this result could not be obtained in the HMM, which does not explicitly describe a potential transition between continuous and intermittent presentation. However, note that this result should be interpreted carefully due to reduced estimation precision of the parameters $b_{S}/{\tilde{b}}_{S}$ and *b*_*S*_/*b*.

In summary, this analysis of the HBM model parameters suggests the following explanation for the observed phenomenon that the alternation rate of the perceived percepts is increased for patients with schizophrenia during intermittent stimulation, while being decreased during continuous stimulation. In general, the relative time spent in the unstable state was increased in patients with schizophrenia. According to the HBM, this was attributed to an increased probability of transition from the stable to the unstable state, which could be potentially related to a decreased recruitment of neurons in the stable state. More specifically, a larger neuronal pool is hypothesized to account for the increased stability, and we accordingly observe a smaller increase in the neuronal pool from continuous to intermittent stable presentation in the patients with schizophrenia, which is suggested by the HBM as a main mechanism for the observed group differences. This analysis of a potential underlying mechanism, explaining the observed group differences also in the transition from continuous to intermittent presentation, is a particular advantage of the HBM over the HMM, because the HBM provides mechanistic explanations and variables with potential neurophysiological interpretations.

## Discussion

### Summary and implications

In the present article we have proposed a model framework for the description and analysis of perceptual responses to bistable stimuli. In particular, the first goal was to describe a high number of observed patterns in responses to continuous and intermittent stimulation and their differences between a group of patients with schizophrenia and healthy controls. The variety of patterns includes more or less regular dominance times during continuous stimulation and a switching between long and short dominance times, i.e., stable and unstable states, during intermittent stimulation, with a tendency for periodically occurring percept changes.

We started on a descriptive level, assuming that dominance times were generated by a simple HMM with only one state for continuous presentation and a stable and an unstable state in intermittent presentation. The HMM was sufficiently small to allow model fit to short empirical data sets and could also describe the high variety of empirically observed response patterns in continuous and intermittent presentation. Interestingly, it also revealed a high degree of reproducibility of response patterns of the same subject across different sessions. In addition, it allowed to relate observed group differences in the rate of percept alternations to HMM parameters, suggesting that especially the relative time spent in the stable state was reduced in the patients with schizophrenia.

Our second goal was to relate the observed response patterns and group differences to potential underlying mechanisms and thus, to build a link to models with detailed neurophysiological assumptions [7, 13, 14, 18, 20] that may not include all types or response patterns and/or may not allow fitting to short data sets. To that end, we proposed a hierarchical model of interacting Brownian motions (HBM). The HBM is based on the common assumption that the sequence of percept changes results from a competition of conflicting neuronal populations [14, 17, 18, 20, 27]. Instead of modeling these in detail, we describe the activity difference by a Brownian motion *P* with drift *ν*_0_ [21] between two borders ±*b*, where the first hitting times of the borders indicate percept changes. Roughly speaking, the drift *ν*_0_ could be considered related to the neuronal interactions within and between the populations, while the border *b* could be considered related to the population sizes. In order to describe responses to intermittent presentation, this mechanism is adapted in another population pair. These populations exhibit a corresponding background process *B* that evokes switching between stable and unstable states, similar to switching between the two percepts. In particular, *B* causes the perception process *P* to change parameters from small drift *ν*_*S*_ and large border *b*_*S*_ in the stable state to fast drift *ν*_*U*_ and small border *b*_*U*_ in the unstable state.

The HBM could be fitted nicely to the given empirical data set, reproducing a high variety of response patterns to continuous and intermittent stimulation in healthy subjects and patients with schizophrenia. In particular, the model fit was even improved over the descriptive HMM by reproducing shorter stable dominance times before a change to the unstable state. The HBM also provided more detailed explanations for the observed group difference that patients with schizophrenia showed higher alternation rates during intermittent stimulation, while percept alternation was decreases during continuous presentation. In particular, the HBM contains additional mechanisms of switching between stable and unstable state for intermittent presentation, which is assumed inactive during continuous presentation. The HBM, similar to the HMM, suggests an increased probability of switching to the unstable state for the patients with schizophrenia and thus, a longer relative time spent in the unstable state. The HBM also provides additional potential explanations related to the borders, or assumed population sizes, suggesting a higher increase from continuous (border *b*) to stable intermittent presentation (border *b*_*S*_) in the healthy subjects. This is a first finding on the transition from continuous to intermittent presentation, which results from including both continuous and intermittent presentation in one model.

These findings suggested by the HBM, which include a longer relative time spent in the unstable state for the patients with schizophrenia and a smaller population size involved in percept stabilization, are also in agreement with recent findings of [41]. They studied the learning behavior of healthy subjects of whom the degree of delusional ideation [42] had been measured. In compliance with earlier studies [for a review see 43], they reported that subjects with larger delusion proneness made decisions on the basis of less information and were also less resilient against irrelevant information [compare also the literature about jumping to conclusions, e.g. 44, 45]. In the present setting, the ambiguous stimulus represents a constant source of partly contradicting visual information [see also 5, 8]. In that sense, the unstable state could be considered a state in which one is less resilient against this contradicting visual information, which yields a high rate of percept changes. The fact that the patients with schizophrenia spent more time in the unstable state is therefore highly consistent with the findings of [41]. Moreover, this finding is also compatible with current models of schizophrenia in the framework of predictive coding [46] that propose a reduced top-down influence of stored predictions. However, it goes beyond previous work by highlighting the role of a background process that controls the balance between stable and unstable states in perceptual inference. In addition, the population sizes could be considered related to the amount of information taken into consideration to create a percept. Again, consistently with [41], we find, in the stable state, larger estimated population sizes, *b*_*S*_, of the perceptual populations *L* and *R* in healthy controls than in patients with schizophrenia. Also, these population sizes are typically much larger than the population sizes in the unstable state (*b*_*S*_ >> *b*_*U*_), which would be consistent with the notion that subjects in the unstable state need less information to change their perception.

### Applicability and model extensions

The HBM may also be used to describe dominance times resulting from other experiments with ambiguous visual stimuli studying, e.g., motion-induced-blindness, binocular rivalry, moving plaids, the Necker Cube, orthogonal gratings or the house/face-paradoxon [e.g. 21, 47] or also bistable auditory stimuli [48]. The HBM is, however, not designed for tristable stimuli, and transient stimulus manipulations as used in after-effect studies cannot be captured by the HBM in its current form. In different bistable settings, the HBM cannot be applied directly, but would allow for potential extensions. For example, in its present form, the HBM describes only balanced perception. However, it could be extended with respect to unbalanced bistable displays, e.g., for different eye contrasts during binocular rivalry [49], by choosing different drift parameters for the positive and the negative drift direction during presentation. Similarly, the drift could be chosen to vary as a function of attention [50, 51] or as a function of long-term history (e.g., the cumulative history *H* proposed in [31]). In studies on mixed perception during binocular rivalry [19], one might use an additional border to define an intermediate range for the perception process in which mixed perception is described.

One should note that the HBMi in its current form is restricted to a duration of blank displays *l*_*b*_ ≤ *l*_*p*_ ⋅ *ν*_0_/*ν*_*S*_. For longer blank displays, the mean drift of *P* during stable states, $\nu_{S}^{*}$, will be negative, yielding no perception change with high probability. However, it would be possible to extend the model accordingly, assuming a temporal evolution in the drift parameters, given corresponding extended empirical observations. In addition, note that the border of the perception process is assumed to be *b* during continuous stimulation and *b*_*S*_ (or *b*_*U*_) during intermittent stimulation. Therefore, an instantaneous change of intermittent to continuous presentation is not yet described. Here, we qualitatively assume that the border jumps very fast from *b* to *b*_*S*_ with the onset of a blank display, while going back slowly during stimulation. A transition from continuous to intermittent presentation would therefore instantly change the response pattern, while a reverse transition would gradually reverse the change back to the one-state process. Quantitative validation and fitting of this assumption would be interesting, but requires corresponding empirical observations, in which the length of the presentation period *l*_*p*_ is varied. This would also allow investigation of potential relations between the HBMc and HBMi parameters and thus, between the mechanisms assumed to underlie the identified group differences.

Concerning the impact of the duration of the blank display *l*_*b*_, two aspects should be discussed. First, the HBM can theoretically reproduce a phenomenon reported earlier in [14]. Conditional that one percept has been present for a short while, the probability of a percept change rises with the blank duration *l*_*b*_. In the HBMi, the same is observed during the unstable state with typically short dominance times: During the unstable state the drift in the blank displays, *ν*_*U*_, is typically larger than the drift *ν*_0_ during stimulation. Therefore, longer blank displays speed up *P*, thereby reducing perceptual stability.

**Fig 19:**
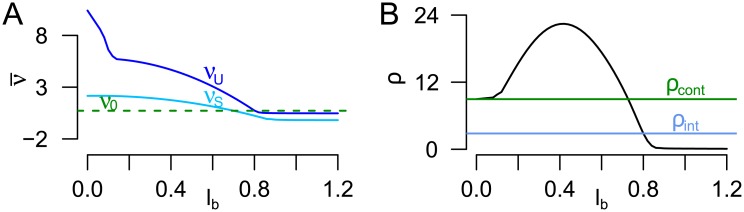
HBMi extension for different lengths of blank display. Parameters derived for subject B and C in Tables 3 and 4, which is the same subject during continuous and intermittent presentation. A) The mean drifts *ν*_*S*_, *ν*_*U*_ decrease with the length of the blank display *l*_*b*_. B) The resulting mean alternation rate *ρ* under intermittent stimulation (black) is derived using formula (18) (Materials and methods). The empirical mean alternation rates per minute of the subject during intermittent viewing with *l*_*b*_ = 0.8 and during continuous viewing are marked by blue and green lines, respectively.

Second, one interesting potential model extension is concerned with the relationship between the length of the blank display and the alternation rate. As reported earlier by [14, 15, 18], the mean dominance time in intermittent presentation has been found to be a function of the relationship between the presentation length *l*_*p*_ (or ‘ON’-period) and the length of the blank display *l*_*b*_ (or ‘OFF’-period). Particularly, the dependence between *l*_*b*_ and the alternation rate is non-monotonic, as would be implied in the HBMi, but follows an inverted U-shape [22, 24, 25] with a peak roughly at 0.4 s. Such an inverted U-shape would be possible in a model extension of the HBMi. As discussed in the Results, the drift terms *ν*_*S*_, *ν*_*U*_ only represent the mean drift across the period of blank display, which is sufficient and parsimonious in the given data set with fixed length of blank display. However, the model would be fully consistent with the assumption that the drifts change during the ‘OFF’-period, such that the mean drifts *ν*_*S*_(*l*_*b*_) and *ν*_*U*_(*l*_*b*_) are functions of the length of the blank display *l*_*b*_. In Fig 19A these mean drifts *ν*_*S*_, *ν*_*U*_ decrease with *l*_*b*_, where the stronger drifts at the beginning of the blank display could be effects of the recent stimulation. Panel B shows the resulting mean alternation rate, which has an inverted U-shape with a maximum around 0.4 s and shows increased stability under intermittent stimulation for *l*_*b*_ > 0.7. This increased stability is caused first by a small drift *ν*_*S*_ < *ν*_0_ in that range. Second, it is also caused by the fact that the time interval *l*_*b*_ in which the background process *B* has positive drift is longer, leading to an increased probability to reach ${\tilde{b}}_{S}$ and thus, to stay in the stable state. Estimation of the functions *ν*_*S*_(*l*_*b*_) and *ν*_*U*_(*l*_*b*_) from a suitable data set with variable lengths of blank displays would be an interesting task.

In summary, the proposed HBM intends to provide a link between empirical data analysis and mechanistic modeling. On the one hand, it aims at precisely describing the high variety of response patterns observed in perceptual responses to bistable stimuli. On the other hand, it aims at bridging the gap to detailed mechanistic models of bistable perception, allowing assumed processes to be fitted to short empirical data sets and thus, also the analysis of group differences. Various extension possibilities show a potential of the HBM to investigate related experimental contexts. By including both continuous and intermittent stimulation, the HBM can thus provide interesting new hypotheses on potential neuronal mechanisms of cognitive phenomena.

## Materials and methods

### Estimation of HMM parameters

Here we describe the estimation procedures of the HMM parameters for continuous presentation and for intermittent presentation. We denote by *d* ≔ (*d*_1_, *d*_2_, …, *d*_*n*_) the set of dominance times modeled as realizations of random variables *D* = (*D*_1_, …, *D*_*n*_).

#### Continuous presentation: ML estimation

The HMM for continuous presentation assumes that all dominance times *d*_*i*_ are independent and Inverse Gaussian distributed with mean *μ* and standard deviation *σ*. The log-likelihood function is then given by

$$\begin{array}{c} \log L\left(d|\mu ,\sigma \right)=\frac{1}{2}\;\sum_{i=1}^{n}\;\log(\frac{\mu^{3}}{2\pi \sigma^{2}d_{i}^{3}})-\frac{\mu }{\sigma^{2}}\frac{{\left(d_{i}-\mu \right)}^{2}}{d_{i}}. \end{array}$$ (12)

Setting the partial derivatives with respect to *μ* and *σ* to zero yields the estimates of Eq (12) [32]:

$$\begin{array}{c} \hat{\mu }={\bar{\mu }}_{d}\;≔\;\frac{1}{n}\;\sum_{i=1}^{n}\;d_{i}\;\text{and}\;\hat{\sigma }=\sqrt{\frac{{\bar{\mu }}_{d}^{3}}{n}\;\sum_{i=1}^{n}\;\left(1/d_{i}-1/{\bar{\mu }}_{d}\right)}. \end{array}$$

#### Intermittent presentation: Baum-Welch-Algorithm

The HMM for intermittent presentation uses the parameter set *ϑ* = (*μ*_*S*_, *σ*_*S*_, *μ*_*U*_, *σ*_*U*_, *p*_*SS*_, *p*_*UU*_) for the mean and standard deviation in the stable and unstable state, respectively, and the transition probabilities between these two states. This parameter set is estimated with the Baum-Welch-Algorithm [BWA, 33] which is an iteratively working instance of the EM-Algorithm maximizing the model likelihood locally. Here, we explain it briefly [for details see, e.g., 34]. In the first step one applies the so called Forward- and Backward-Algorithm. The forward-variable *α*_*j*_(*i*) is defined as the probability of observing the sequence *d*_1_, *d*_2_, …, *d*_*i*_ and being in state *j* at time *i*, given the model parameters. The backward-variable *β*_*j*_(*i*) denotes the analogous probability of observing the ending partial sequence *d*_*i*+1_, *d*_*i*+2_, …, *d*_*n*_ and being in state *j* at time *i*. To avoid underflow we normalize both variables [e.g. 34], resulting in the normalized variables ${\tilde{\alpha }}_{j}\left(i\right)$, ${\tilde{\beta }}_{j}\left(i\right)$. The normalized variables can be derived iteratively as follows

$$\alpha_{j}^{*}\left(1\right)\;≔\;\pi_{j}f_{\mu_{j},\sigma_{j}}^{\text{IG}}\left(d_{1}\right),\;\;c_{i}\;≔\;\alpha_{S}^{*}\left(i\right)+\alpha_{U}^{*}\left(i\right),\;\;{\tilde{\alpha }}_{j}\left(i\right)\;≔\;\alpha_{j}^{*}\left(i\right)/c_{i},\;\;\alpha_{j}^{*}\left(i\right)\;≔\;f_{\mu_{j},\sigma_{j}}^{\text{IG}}\left(d_{i}\right)\;\sum_{k\in \{S,U\}}\;{\tilde{\alpha }}_{k}\left(i-1\right){\tilde{p}}_{kj}\;\text{for}\;i=2,\ldots ,n,$$

$${\tilde{\beta }}_{j}\left(n\right)=1/c_{n}\;\text{and}\;{\tilde{\beta }}_{j}\left(i\right)=\sum_{k\in \{S,U\}}\;p_{jk}f_{\mu_{k},\sigma_{k}}^{\text{IG}}\left(d_{i+1}\right){\tilde{\beta }}_{k}\left(i+1\right)/c_{i}\;\text{for}\;i=n-1,\ldots ,1,$$

where *π*_*j*_ denotes the probability that the Markov chain starts in state *j*, *p*_*SU*_ = 1 − *p*_*SS*_, *p*_*US*_ = 1 − *p*_*UU*_ and $f_{\mu ,\sigma }^{\text{IG}}\left(x\right)$ denotes the density of the IG distribution with expectation *μ* and standard deviation *σ* evaluated at *x*. Note that we suppress the dependence of ${\tilde{\alpha }}_{j}\left(i\right)$ and ${\tilde{\beta }}_{j}\left(i\right)$ on the parameter set *ϑ* for convenience.

The forward and backward variables are used to derive the probability *γ*_*j*_(*i*|*ϑ*) of being in state *j* at time *i*, given the whole sequence *d* ≔ (*d*_1_, …, *d*_*n*_) and the parameters *ϑ*

$$\begin{array}{c} \gamma_{j}\left(i|\vartheta \right)=\frac{{\tilde{\alpha }}_{j}\left(i\right){\tilde{\beta }}_{j}\left(i\right)}{{\tilde{\alpha }}_{S}\left(i\right){\tilde{\beta }}_{S}\left(i\right)+{\tilde{\alpha }}_{U}\left(i\right){\tilde{\beta }}_{U}\left(i\right)}. \end{array}$$

Moreover, we need the probability *ξ*_*j*,*k*_(*i*|*ϑ*) of being in state *j* at time *i* and in state *k* at time *i* + 1, given the data *d* and the parameters *ϑ*,

$$\begin{array}{c} \xi_{j,k}\left(i|\vartheta \right)=\frac{{\tilde{\alpha }}_{j}\left(i\right)p_{jk}{\tilde{\beta }}_{k}\left(i+1\right)f_{\mu_{k},\sigma_{k}}^{\text{IG}}\left(d_{i+1}\right)}{\sum_{j}\sum_{k}{\tilde{\alpha }}_{j}\left(i\right)p_{jk}{\tilde{\beta }}_{k}\left(i+1\right)f_{\mu_{k},\sigma_{k}}^{\text{IG}}\left(d_{i+1}\right)}. \end{array}$$

To iteratively derive the parameter estimates, the BWA applies expectation maximization as follows. Let *ϑ*^(*m*)^ denote the parameter estimates after the *m*-th iteration step, and let Y denote the set of all possible state sequences of the hidden Markov chain. Let *Y* = (*Y*_1_, …, *Y*_*n*_) denote a Y-valued random variable and *y* = (*y*_1_, …, *y*_*n*_) a realization of *Y*. The BWA then iteratively maximizes the *Q-function* [e.g. 35] over *Y*,

$$Q\;≔\;Q(\vartheta |\vartheta^{\left(m\right)})=\sum_{y\in Y}\;\log L\left(d,y|\vartheta \right)P\left(Y=y|d,\vartheta^{\left(m\right)}\right),$$

i.e., the expectation of the complete-data log-likelihood *L* across all possible paths y∈Y. The updated parameter set *ϑ*^(*m*+1)^ is chosen such that it maximizes *Q*. For a fixed state sequence *y* = (*y*_1_, …, *y*_*n*_) the log-likelihood of the data is

$$\begin{array}{c} \log L\left(d,y|\vartheta \right)=\log\pi_{y_{1}}+\log f_{\mu_{y_{1}}\sigma_{y_{1}}}^{\text{IG}}\left(d_{1}\right)+\;\sum_{i=2}^{n}\;(\log\left(p_{y_{i-1}y_{i}}\right)+\log\left(f_{\mu_{y_{i}},\sigma_{y_{i}}}^{\text{IG}}\left(d_{i}\right)\right)). \end{array}$$

Insertion into *Q* yields

$$\begin{array}{ccc} Q(\vartheta |\vartheta^{\left(m\right)}) & = & \sum_{y_{1}\in \left\{S,U\right\}}\;\log\pi_{j}P\left(Y_{1}=y_{1}|d,\vartheta^{\left(m\right)}\right)\\ & + & \sum_{y_{i-1}\in \left\{S,U\right\}}\;\sum_{y_{i}\in \left\{S,U\right\}}\;\sum_{i=2}^{n}\;\log p_{y_{i-1}y_{i}}P\left(Y_{i-1}=y_{i-1},Y_{i}=y_{i}|d,\vartheta^{\left(m\right)}\right)\\ & + & \sum_{y_{i}\in \left\{S,U\right\}}\;\sum_{i=1}^{n}\;\frac{1}{2}P\left(Y_{i}=y_{i}|d,\vartheta^{\left(m\right)}\right)\;(\log(\frac{\mu_{y_{i}}^{3}}{2\sigma_{y_{i}}^{2}\pi d_{i}^{3}})-\frac{\mu_{y_{i}}}{\sigma_{y_{i}}^{2}}\frac{{\left(d_{i}-\mu_{y_{i}}\right)}^{2}}{d_{i}}). \end{array}$$

Note that the first line depends only on the initial distribution *π*, the second line depends on the transition probabilities and the third line depends on the parameters of the Inverse Gaussian distributions. Therefore, iterative parameter estimation separately maximizes these terms. Note further that we can rewrite $P\left(Y_{i}=y_{i}|d,\vartheta^{\left(m\right)}\right)=\gamma_{y_{i}}\left(i|\vartheta^{m}\right)$ and $P\left(Y_{i-1}=y_{i-1},Y_{i}=y_{i}|d,\vartheta^{\left(m\right)}\right)=\xi_{y_{i-1}y_{i}}\left(i-1|\vartheta^{m}\right)$, which yields the following estimates in the *m* + 1-st iteration step.

For the entries of the transition matrix, Lagrange maximization of the second line under the constraints *p*_*SS*_ + *p*_*SU*_ = *p*_*UU*_ + *p*_*US*_ = 1 yields the estimate

$$\begin{array}{c} {\hat{p}}_{jk}^{\left(m+1\right)}=\frac{\sum_{i=1}^{n-1}\xi_{j,k}\left(i|\vartheta^{\left(m\right)}\right)}{\sum_{i=1}^{n-1}\gamma_{j}\left(i|\vartheta^{\left(m\right)}\right)}, \end{array}$$

in the (*m* + 1)-th step of the BWA [e.g. 34].

The big bracket in the last line of the *Q*-function is analogous to the likelihood function of the IG distribution (Eq (12)) given in the section ‘Continuous presentation: ML estimation’ (with the additional indices *y*_*i*_ and the weighting factors $P(Y_{i}=y_{i}|d,\vartheta^{\left(m\right)})$). Therefore, setting the partial derivatives to zero yields for *j* ∈ {*S*, *U*} the analogous estimates

$$\begin{array}{c} {\hat{\mu }}_{j}^{\left(m+1\right)}=\frac{\sum_{i=1}^{n}\;\gamma_{j}\left(i|\vartheta^{\left(m\right)}\right)d_{i}}{\sum_{i=1}^{n}\;\gamma_{j}\left(i|\vartheta^{\left(m\right)}\right)},\;\;{\hat{\sigma }}_{j}^{\left(m+1\right)}=\sqrt{\frac{{\hat{\mu }}_{j}^{3}}{\sum_{i=1}^{n}\;\gamma_{j}\left(i|\vartheta^{\left(m\right)}\right)}\sum_{i=1}^{n}\;\gamma_{j}\left(i|\vartheta^{\left(m\right)}\right)(\frac{1}{d_{i}}-\frac{1}{{\hat{\mu }}_{j}})}. \end{array}$$

which are the updates for parameters of the Inverse Gaussian distributions in the (*m* + 1)-th step of the BWA.

In order to update the starting distribution, we do not maximize the respective summand of *Q* but we assume the stationary distribution as the starting distribution *π*. The stationary distribution is derived using the eigenvectors of the transpose of the transition matrix [52], which yields

$$\begin{array}{c} \pi_{S}^{\left(m+1\right)}=\frac{p_{UU}^{\left(m+1\right)}-1}{p_{SS}^{\left(m+1\right)}+p_{UU}^{\left(m+1\right)}-2},\;\text{and}\;\pi_{U}^{\left(m+1\right)}=1-\pi_{S}^{\left(m+1\right)}. \end{array}$$

These iterative steps are repeated until a desired level of convergence is reached.

*Starting values and constraints*. As starting values $\mu_{S}^{\left(s\right)}$, $\sigma_{S}^{\left(s\right)}$, $\mu_{U}^{\left(s\right)}$, $\sigma_{U}^{\left(s\right)}$, $p_{SS}^{\left(s\right)}$, $p_{UU}^{\left(s\right)}$ for the Baum-Welch algorithm we chose, in correspondence with the data set, $p_{SS}^{\left(s\right)}=p_{UU}^{\left(s\right)}=0.5$; $\mu_{S}^{\left(s\right)}=4$; $\;\sigma_{U}^{\left(s\right)}=5$. In order to reduce the probability that the Baum-Welch-Algorithm will be captured in a local extremum, we chose ten equidistant values for $\mu_{S}^{\left(s\right)}$ ranging between 60 and 0.95max_*i*_ *d*_*i*_, and for each value of $\mu_{S}^{\left(s\right)}$ we choose ten equidistant values for $\sigma_{S}^{\left(s\right)}$ between 10 and $1.1\mu_{S}^{\left(s\right)}$. Out of the resulting one hundred sets of parameter estimates we chose the parameter set with the highest log-likelihood. If the response pattern shows only dominance times larger than 30 seconds we reduce the model to the stable phase. The parameters *μ*_*S*_ and *σ*_*S*_ are derived by ML as described in the section ‘Continuous presentation: ML estimation’, and we set *p*_*SS*_ ≔ 1. If the dominance time are only smaller than 30 seconds, we only estimate *μ*_*U*_ and *σ*_*U*_ by ML and use *p*_*UU*_ = 1.

For subjects with relatively clear distinction between long and short dominance times this procedure yields reasonable estimates. For subjects with less clear distinction, we added the following constraints based on the idea that short dominance times should not affect estimation of the stable parameters and long dominance times should not affect estimation of unstable parameters. Note that in continuous presentation where no state exists, about 90% of the dominance times are shorter than 15 seconds, while only about two percent are larger than 30 seconds. Therefore, we first require ${\hat{\sigma }}_{S}>1$ such that not just the largest dominance time is estimated as stable and all others are categorized as unstable (which may increase the likelihood). Second, we do not accept HMMs with ${\hat{\mu }}_{S}<0.98{\hat{\mu }}_{15}$ where ${\hat{\mu }}_{15}\;≔\;(1/|d1_{d>15}\left|\right)\;\sum_{i=1}^{n}\;d_{i}1_{d_{i}>15}$. This prevents dominance times smaller than 15 seconds to be considered for the estimation of *μ*_*S*_. Third, we require ${\hat{\mu }}_{S}<1.02{\hat{\mu }}_{75}$ where ${\hat{\mu }}_{75}\;≔\;(1/|d1_{d>75}\left|\right)\;\sum_{i=1}^{n}\;d_{i}1_{d_{i}>75}$ if any dominance time is larger than 75 seconds and ${\hat{\mu }}_{75}=75$ otherwise such that rather stable dominance times longer than 75 seconds are not classified as unstable.

#### Expected relative time spent in the stable state

Here we derive the formula for the expected time *φ*_*S*_ spent in the stable state investigated in Fig 9. To that end, let *N*_*j*_, *j* ∈ {*S*, *U*}, denote the (random) number of dominance times in state *j* before a state change. As the number of dominance times before a state change is geometrically distributed with probability 1 − *p*_*jj*_, we have for the expectation

$$\begin{array}{c} E\left[N_{j}\right]=\frac{1}{1-p_{jj}}. \end{array}$$

Moreover, let ${\left(D_{i}^{S}\right)}_{i\geq 1}$ be a sequence of independent IG(*μ*_*S*_, *σ*_*S*_)-distributed random variables and ${\left(D_{i}^{U}\right)}_{i\geq 1}$ be a sequence of IG(*μ*_*U*_, *σ*_*U*_)-distributed random variables. We derive *φ*_*S*_ as

$$\begin{array}{cc} \varphi_{S} & ≔\frac{E[\text{length}\;\text{of}\;\text{a}\;\text{stable}\;\text{phase}]}{E[\text{length}\;\text{of}\;\text{a}\;\text{stable}\;\text{phase}+\text{length}\;\text{of}\;\text{an}\;\text{unstable}\;\text{phase}]}. \end{array}$$

The length of a stable phase is a random variable distributed like $\sum_{i=1}^{N_{S}}\;D_{i}^{S}$, where $D_{1}^{S},\ldots ,D_{N_{S}}^{S}$ are independent from each other and also independent of *N*_*S*_. Therefore we have [53]

$$\begin{array}{c} E\;[\sum_{i=1}^{N_{S}}\;D_{i}^{S}]=E[N_{S}]E[D_{i}^{S}]=\frac{\mu_{S}}{1-p_{SS}}, \end{array}$$

and analogously for the expected length of an unstable phase. This yields

$$\varphi_{S}=\frac{\mu_{S}/\left(1-p_{SS}\right)}{\mu_{S}/\left(1-p_{SS}\right)+\mu_{U}/\left(1-p_{UU}\right)}=\frac{\left(1-p_{UU}\right)\mu_{S}}{\left(1-p_{UU}\right)\mu_{S}+\left(1-p_{SS}\right)\mu_{U}}.$$ (13)

### Estimation of HBM parameters

Here we describe the estimation procedures of the HBM parameters for continuous presentation and for intermittent presentation.

#### HBMc

Recall that the HBMc describes the perception process *P* as a Brownian motion with drift ±*ν*_0_, where the sign of the drift changes at the first hitting time of the borders ±*b*. The two parameters (*b*, *ν*_0_) are estimated using the ML method. Due to transformation invariance of the ML estimates, we can therefore use the ML estimators from the section ‘Continuous presentation: ML estimation’ applying that the resulting dominance times are IG distributed with expectation *μ* = 2*b*/*ν*_0_ and variance $\sigma^{2}=2b/\nu_{0}^{3}$, or conversely, $b=\left(1/2\right)\;\cdot \;\sqrt{\mu^{3}/\sigma^{2}}$ and ${\hat{\nu }}_{0}=\sqrt{\mu /\sigma^{2}}$. This yields

$${\hat{\nu }}_{0}=2n\hat{b}/\sum_{i=1}^{n}\;d_{i}\;\text{and}\;\hat{b}=\frac{1}{2}\sqrt{\frac{1}{1/n\sum_{i=1}^{n}\;1/d_{i}-n/\sum_{i=1}^{n}\;d_{i}}}.$$

#### HBMi

The parameters of the HBMi are $\Theta =(b_{S},\nu_{S}^{*},b_{U},\nu_{U}^{*},{\tilde{b}}_{S},{\tilde{b}}_{U},\nu_{B}^{*})$ where $\left(b_{S},\nu_{S}^{*}\right)$ and $\left(b_{U},\nu_{U}^{*}\right)$ denote the border and drift parameters of the perception process *P* in the stable and the unstable state, respectively. ${\tilde{b}}_{S}$, ${\tilde{b}}_{U}$, $\nu_{B}^{*}$ are the border and drift parameters of the background process *B* which determines the hidden state. Recall that the likelihood of the whole model given the data and the parameter vector Θ is approximately

$$\begin{array}{c} L\left(d|\Theta \right)\approx \alpha_{S}\left(n\right)+\alpha_{U}\left(n\right), \end{array}$$

using the forward variables *α*_*j*_(*i*) defined recursively in Eq (10). Note again that we suppress dependence on the parameters Θ for convenience. In practice we need to avoid underflow when calculating *α*_*j*_(*i*). To that end the forward variables are normalized such that ∑_*j*_ *α*_*j*_(*i*) = 1 for all time points *i*, using the following steps [40]. For *j* ∈ {*S*, *U*},

$$\alpha_{j}^{*}\left(1\right)\;≔\;\alpha_{j}\left(1\right),\;\;c_{i}\;≔\;\alpha_{S}^{*}\left(i\right)+\alpha_{U}^{*}\left(i\right),\;\;{\tilde{\alpha }}_{j}\left(i\right)\;≔\;\alpha_{j}^{*}\left(i\right)/c_{i},\;\;\alpha_{j}^{*}\left(i\right)\;≔\;f_{\mu_{j},\sigma_{j}}^{\text{IG}}\left(d_{i}\right)\sum_{k\in \{S,U\}}\;{\tilde{\alpha }}_{k}\left(i-1\right){\tilde{p}}_{kj},\;i>1.$$

The likelihood then derives as

$$\begin{array}{c} L\left(d|\Theta \right)\approx \prod_{i=1}^{n}\;c_{i}\left({\tilde{\alpha }}_{S}\left(n\right)+{\tilde{\alpha }}_{U}\left(n\right)\right)=\prod_{i=1}^{n}\;c_{i},\;\text{yielding}\;\log L\approx \;\sum_{i=1}^{n}\;\log(c_{i}). \end{array}$$ (14)

Parameter estimation is then obtained by maximizing ∑_*i*_ log(*c*_*i*_), which is a function of the model parameters Θ. To that end we apply the Newton-type algorithm [54] implemented in the R-function nlm(). Alternatively the COBYLA-Algorithm [55] for maximization under non-linear constraints can be applied.

Next we discuss the set of starting values $\left\{b_{S}^{\left(s\right)},{\nu_{S}^{*}}^{\left(s\right)},b_{U}^{\left(s\right)},{\nu_{U}^{*}}^{\left(s\right)},{\tilde{b}}_{S}^{\left(s\right)},{\tilde{b}}_{U}^{\left(s\right)},{\nu_{B}^{*}}^{\left(s\right)}\right\}$ for the optimization algorithm. Let $U\;≔\;\{{\hat{\mu }}_{15},{\hat{\mu }}_{30}\}$ and $O\;≔\;\{{\hat{\sigma }}_{15},{\hat{\sigma }}_{30},1.15{\hat{\sigma }}_{30}\}$, where ${\hat{\mu }}_{k}$ and ${\hat{\sigma }}_{k}$ denote the empirical mean and standard deviation of all dominance times larger than *k* seconds. Then we choose the initial values for *b*_*S*_ and $\nu_{S}^{*}$ from the sets

$$\begin{array}{c} b_{S}^{\left(s\right)}\in \left\{\sqrt{\mu^{3}/\sigma^{2}}/2|\mu \in U,\sigma \in O\right\},\;\;{\nu_{S}^{*}}^{\left(s\right)}\in \left\{\sqrt{\mu /\sigma^{2}}|\mu \in U,\sigma \in O\right\}. \end{array}$$

Depending on ${\nu_{S}^{*}}^{\left(s\right)}$ and $b_{S}^{\left(s\right)}$, we choose $b_{U}^{\left(s\right)}\in \left\{0.01b_{S}^{\left(s\right)},0.05b_{S}^{\left(s\right)},0.15b_{S}^{\left(s\right)}\right\}$, ${\tilde{b}}_{S}^{\left(s\right)}\in \left\{b_{S}^{\left(s\right)},10b_{S}^{\left(s\right)}\right\}$, $\nu_{U}^{*}{}^{\left(s\right)}\in \left\{1.01\nu_{S}^{*\left(s\right)},3\nu_{S}^{*\left(s\right)},7\nu_{S}^{*\left(s\right)}\right\}$, furthermore ${\nu_{B}^{*}}^{\left(s\right)}\in \left\{0.1,3\right\}$ and ${\tilde{b}}_{U}^{\left(s\right)}\in \left\{-3,0,3\right\}$.

The initial value for $\pi_{S}=P$(process starts in the stable state) is set to ${\hat{\pi }}_{S}=1$ if *d*_1_ ≥ 45, ${\hat{\pi }}_{S}=0$ if *d*_1_ ≤ 15, and ${\hat{\pi }}_{S}=1/2$ otherwise. Alternatively and comparable to the HMM the stationary distribution can be used for the initial distribution, thereby reducing the number of parameters by one. As this, however, requires numerical integration and increases the computational effort considerably and the differences between the two approaches were negligible we used the simpler rule.

The maximization algorithm is applied using all combinations of starting values. We then take the set of parameter estimates which yields the highest log-likelihood and fulfills the following constraints

$$\text{A)}\;\nu_{U}\geq \nu_{S};\;\text{B)}\;0\leq b_{U}\leq b_{S};\;\text{C)}\;{\tilde{b}}_{S}\geq 0;\;\text{D)}\;\nu_{B}>0;\;\text{E)}\;\mu_{S}^{*}\approx 2b_{S}/\nu_{S}^{*}\geq 0.98{\hat{\mu }}_{15};\;\text{F)}\;\sigma_{S}^{*}\approx \sqrt{2b_{S}/{\nu_{S}^{*}}^{3}}<1.20{\hat{\sigma }}_{15}.$$

Constraints A) to D) result from the model assumptions, E) prevents dominance times smaller than 15 seconds to be considered for the estimation of *μ*_*S*_, analogously to the HMM procedure. Constraint F) prevents too big estimates of standard deviations, which would yield implausible results.

In cases in which only dominance times larger than 30 seconds are observed, we apply the algorithm described for the HBMc, where *b_S_*, $\nu_{S}^{*}$ are estimated like in the section ‘HBMc’ and ${\tilde{b}}_{S}$ is set to zero. In cases in which only dominance times up to 30 seconds are observed, we proceed analogously, where *b_U_*, $\nu_{U}^{*}$ are estimated like in the section ‘HBMc’ and set ${\tilde{b}}_{U}=10^{10}$. In either case we set $\nu_{b}^{*}=10$ and do not estimate the other variables.

### HBMi: Dominance time before state changes

#### Stable dominance time before a change to the unstable state

In the section ‘Relation of the HBMi to the two state HMM’ we discussed that in the HBMi, stable dominance times are shorter when occurring directly before a state change to the unstable state than when followed by another stable dominance time. The same observation was also made in the sample dataset (Fig 17). Therefore, we derive here the expected length $\mu_{S}^{-}$ of a stable dominance time before a state change and its corresponding expected length $\mu_{S}^{+}$ before another stable dominance time.

Let $\Theta =(b_{S},\nu_{S}^{*},b_{U},\nu_{U}^{*},{\tilde{b}}_{S},{\tilde{b}}_{U},\nu_{B}^{*})$ be the parameter set of an HBMi, and let $D_{1}^{S}$ denote an $\text{IG}(2b_{S}/\nu_{S}^{*},\sqrt{2b_{S}/{\nu_{S}^{*}}^{3}})$-distributed random stable dominance time. Further, let $p_{SS}^{*}$ denote the probability to stay in the stable state (8), and let *Y*_*i*_ denote the hidden state during the *i*-th dominance time. Then it holds

$$\begin{array}{ccc} \mu_{S}^{+} & ≔ & E\left[D_{1}^{S}|Y_{1}=S,Y_{2}=S\right]=E\left[D_{1}^{S}|Y_{1}=S,B_{D_{1}^{S}}\geq {\tilde{b}}_{S}\right]\\ & = & \frac{\int_{0}^{\infty }P(D_{1}^{S}=t,B_{D_{1}^{S}}\geq {\tilde{b}}_{S})tdt}{P(B_{D_{1}^{S}}\geq {\tilde{b}}_{S})}. \end{array}$$

Now we integrate across all possible values *t* that $D_{1}^{S}$ can take, using independence of *B* and *P* during $D_{1}^{S}$, IG distribution of $D_{1}^{S}$ and normal distribution of $B_{D_{1}^{S}}$. Further we note that the probability in the denominator equals $p_{SS}^{*}$, which yields

$$\mu_{S}^{+}={\left(p_{SS}^{*}\right)}^{-1}\int_{0}^{\infty }\;f_{2b_{S}/\nu_{S}^{*},\sqrt{2b_{S}/{\nu_{S}^{*}}^{3}}}^{\text{IG}}\left(t\right)\left(1-\Phi_{\nu_{B}^{*}t,t}\left({\tilde{b}}_{S}\right)\right)tdt.$$ (15)

Analogously, we obtain

$$\mu_{S}^{-}=E[D_{1}^{S}|Y_{1}=S,Y_{2}=U]={\left(1-p_{SS}^{*}\right)}^{-1}\int_{0}^{\infty }f_{2b_{S}/\nu_{S}^{*},\sqrt{2b_{S}/\nu_{S}^{*}{}^{3}}}^{\text{IG}}\left(t\right)\Phi_{\nu_{B}^{*}t,t}({\tilde{b}}_{S})tdt.\;$$ (16)

#### Expected relative time spent in the stable state

In this section we derive the formula for the expected relative time $\varphi_{S}^{*}$ spent in the stable state in the HBMi analyzed in the section ‘Application of the HBM to the sample data set’. To that end, let $N_{j}^{*}$, *j* ∈ {*S*, *U*}, denote the (random) number of dominance times of the HBMi in state *j* before a state change. As the number of dominance times before a state change is geometrically distributed with probability $1-p_{jj}^{*}$ its expectation is given by $E\left[N_{j}^{*}\right]={\left(1-p_{jj}^{*}\right)}^{-1}$. (Again, due to taking the mean of different drifts, this derivation is only a close approximation, but we omit approximation signs here for convenience.) Again, we derive $\varphi_{S}^{*}$ as

$$\varphi_{S}^{*}\;≔\;\frac{E[\text{length}\;\text{of}\;\text{a}\;\text{stable}\;\text{phase}]}{E[\text{length}\;\text{of}\;\text{a}\;\text{stable}\;\text{phase}+\text{length}\;\text{of}\;\text{an}\;\text{unstable}\;\text{phase}]}.$$

The length of a stable phase is a random variable distributed like $\sum_{i=1}^{N_{S}^{*}}\;D_{i}^{S}$, where $D_{1}^{S},\ldots ,D_{N_{S}^{*}}^{S}$ denote the random stable dominance times. Given $N_{S}^{*}$, we use linearity of expectation to compute the conditional expectation

$$E\;[\sum_{i=1}^{N_{S}^{*}}\;D_{i}^{S}|N_{S}^{*}]=\left(N_{S}^{*}-1\right)\mu_{S}^{+}+\mu_{S}^{-}.$$

Now we take expectation over $N_{S}^{*}$ to find

$$E\;[\sum_{i=1}^{N_{S}^{*}}\;D_{i}^{S}]=\frac{p_{SS}^{*}}{1-p_{SS}^{*}}\mu_{S}^{+}+\mu_{S}^{-}.$$

An analogous result holds for the expected length of an unstable phase. This yields

$$\begin{array}{c} \varphi_{S}^{*}=\frac{\frac{p_{SS}^{*}}{1-p_{SS}^{*}}\mu_{S}^{+}+\mu_{S}^{-}}{\frac{p_{SS}^{*}}{1-p_{SS}^{*}}\mu_{S}^{+}+\mu_{S}^{-}+\frac{p_{UU}^{*}}{1-p_{UU}^{*}}\mu_{U}^{+}+\mu_{U}^{-}}. \end{array}$$ (17)

### HBMi: Derivation of the alternation rate *ρ* as used in Fig 19B

Here, we derive a formula for the alternation rate *ρ* used in Fig 19 given the HBMi parameter set $\left(\mu_{S}^{*},\sigma_{S}^{*},\mu_{U}^{*},\sigma_{U}^{*},p_{SS}^{*},p_{UU}^{*}\right)$. For each length of blank display *l*_*b*_ and the value of *ν*_*S*_, *ν*_*U*_ as shown in Fig 19A the mean drifts per second in the stable and the unstable state $\nu_{S}^{*}$, $\nu_{U}^{*}$ and the mean drift of the background process $\nu_{B}^{*}$ are derived using Eq (4). Then, we use Eqs (8) and (11) to derive the values $\left(\mu_{S}^{*},\sigma_{S}^{*},\mu_{U}^{*},\sigma_{U}^{*},p_{SS}^{*},p_{UU}^{*}\right)$ given the mean drifts per second, and we recall Eq (17) for $\varphi_{S}^{*}$, and analogously for $\varphi_{U}^{*}$

We now show that the alternation rate can be described as

$$\begin{array}{c} \rho \;≔\;\lim_{\Delta \to \infty }\frac{E[N^{*}\left(\Delta \right)]}{\Delta }=\frac{\varphi_{S}^{*}}{\mu_{S}^{*}}+\frac{\varphi_{U}^{*}}{\mu_{U}^{*}}, \end{array}$$ (18)

where N*(Δ) denotes the number of perceptual changes in an interval of length Δ. We split up $N^{*}\left(\Delta \right)=N_{S}^{*}\left(\Delta \right)+N_{U}^{*}\left(\Delta \right)$, where $N_{S}^{*}\left(\Delta \right)$ and $N_{U}^{*}\left(\Delta \right)$ denote the number of perceptual changes in the respective stable and unstable phases in the interval of length Δ. We then show

$$\begin{array}{c} \frac{E[N_{S}^{*}\left(\Delta \right)]}{\Delta }\underset{\Delta \to \infty }{\to }\frac{\varphi_{S}^{*}}{\mu_{S}^{*}}, \end{array}$$

and analogously for the unstable state. To that end, let Δ_*S*_ be the time spent in the stable state in a time interval of length Δ. By the Elementary Renewal Theorem [e.g. 56] it holds

$$\begin{array}{c} \frac{E[N_{S}^{*}\left(\Delta \right)]}{\Delta_{S}}=\frac{E[N_{S}^{*}\left(\Delta_{S}\right)]}{\Delta_{S}}\underset{\Delta \to \infty }{\to }\frac{1}{\mu_{S}^{*}} \end{array}$$

as Δ → ∞ naturally implies Δ_*S*_ → ∞. According to the definition of $\varphi_{S}^{*}$ as the expected relative time spent in the stable state, we get $\Delta_{S}/\Delta \to \varphi_{S}^{*}$ in probability. This yields the claim

$$\begin{array}{c} \frac{E[N_{S}^{*}\left(\Delta \right)]}{\Delta }=\frac{\Delta_{S}}{\Delta }\cdot \frac{E[N_{S}^{*}\left(\Delta \right)]}{\Delta_{S}}\underset{\Delta \to \infty }{\to }\frac{\varphi_{S}^{*}}{\mu_{S}^{*}}. \end{array}$$

### Experimental protocol and data preprocessing

#### Details on experimental protocol

The sample data set for main analysis was collected as described in [10], the sample data for the analysis of reproducibility was collected as described in [9]. In both cases, participants took part in a behavioral experiment including continuous and intermittent stimulation with the same ambiguous stimulus. This stimulus was a structure-from-motion stimulus that appears as a rotating sphere. The rotation direction of the sphere is ambiguous and equally compatible with leftward and rightward percepts. For continuous stimulation, the sphere was presented throughout four minutes in which participants’ perception spontaneously alternated between the two percepts. Participants indicated perceptual alternations via button presses on a keyboard. For intermittent stimulation, the sphere was presented for twenty minutes repeatedly for short intervals of 0.6 s interleaved by blank screens of 0.8 s duration. Here, participants indicated the perceived rotation direction at every 1.4 s at each presentation of the sphere. Please refer to [9, 10] for a detailed description of the data collection. As suggested in [10], missing responses during intermittent stimulation were replaced by their preceding responses because the reported percept typically persisted to the next available response.

#### Investigation of history dependence in the response patterns

Long-term dependencies in the data set of [10] during continuous presentation are analyzed using the Pearson correlation coefficient *c*_*H*_ between the dominance times and the cumulative history *H* as introduced in [31]. The history *H* is a function of the length and recency of previously dominated percepts. For each of the 57 subjects with at least five dominance times, *c*_*H*_ is estimated as explained in [31]. To assess statistical significance, 1000 data sets are obtained for each subject by permutation of the dominance times to approximate the distribution of *c*_*H*_ under the null hypothesis of independent and identically distributed dominance times. Statistical significance on the 5% level is obtained by comparison of the empirical history *c*_*H*_ to the 95% quantile of the distribution of *c*_*H*_ derived from the permuted data sets.

## Supporting information

S1 Data Raw data including dominance times from the studies [9, 10]. Data include dominance times for intermittent (AlbertetalDataINT2015.RData) and continuous (AlbertetalDataCON2015.RData) stimulation for each of the 61 subjects of [10], and dominance times of two trials of continuous stimulation for each of the 105 subjects of [9] (AlbertetalDataCON2013.RData). A pdf file (AlbertetalDataDescription.pdf) provides a detailed description of the datasets. The data files and pdf file are packed in the supporting information file S1_Data.zip.
(ZIP)
