# Supplementary material for: A hierarchical stochastic model for bistable perception
Source: PLoS Comput Biol. 2017 Nov 20;13(11):e1005856. doi: 10.1371/journal.pcbi.1005856 (PMC5714404; doi:10.1371/journal.pcbi.1005856)
Supplement: S1 Data — Data include dominance times for intermittent (AlbertetalDataINT2015.RData) and continuous (AlbertetalDataCON2015.RData) stimulation for each of the 61 subjects of [10], and dominance times of two trials of continuous stimulation for each of the 105 subjects of [9] (AlbertetalDataCON2013.RData). A pdf file (AlbertetalDataDescription.pdf) provides a detailed description of the datasets. The data files and pdf file are packed in the supporting information file S1_Data.zip. (ZIP) [file pcbi.1005856.s001.zip › Data/AlbertetalDataDescription.pdf]

Data of Schmack et al 2015 Schizophr Res Cog 2(2):72-77

Stimulus: continuous/intermittent presentation of bistable rotating sphere

Subjects: 61 subjects: 29 patients with schizophrenia and 32 controls
In all data 'Left' is coded as 97 and 'Right' as 98. Other numbers indicate unclear perception.

Continuous presentation

Filename: AlbertetalCon2015.RData with **variables**

- `daten.2015con`: List with 61 elements each being a data frame with the start and end of the experiment in the first column, pressed button as second column and the time of button presses as third column (unit: millisecond). Attention: Subjects may have pressed the same button subsequently.
- `dom.2015con`: List with 61 elements each being a vector of the dominance times (in seconds) of the corresponding subject
- `group`: vector of 68 entries. 0, if patient with schizophrenia, 1, if control subject, NA if no data available
- `group_s`: vector containing the numbers of the patients with schizophrenia
- `group_c`: vector containing the numbers of the controls
- `person2015`: vector containing the number of the subjects with data

Last update: 24.07.2017 by Stefan Albert

Intermittent presentation

Filename: AlbertetalInt2015.RData with **variables**

- `daten.2015int`: List with 61 elements each being a data frame with the trial number as first column, pressed button as second column, the time of stimulus onset as fourth column, the time of button presses as fivth column and the reaction time (difference between fivth and fourth column) as sixth column (unit: millisecond).
- `dom.2015int`: List with 61 elements each being a vector of the dominance times of the corresponding subject in seconds
- `dom.2015int.trials`: List with 61 elements each being a vector of the dominance times of the corresponding subject in number of trials
- `perc.2015int`: List with 61 elements each being a vector with the perceived percept in each of the 860 trials (contradicting answers during one trial are declared as missing data)
- `perc.2015int.re`: List with 61 elements each being a vector with the perceived percept in each of the 860 trials. Here missing data are substituted by their predecessor.
- `group`, `group_s`, `group_c`, `person2015`: like in the continuous case

Last update: 24.07.2017 by Stefan Albert

Data of Schmack et al 2013 J of Neurosci 33(34):13701-12

Stimulus: continuous presentation of bistable rotating sphere

Subjects: 105 healthy subjects
In all data 'Left' is coded as 97 and 'Right' as 98. Other numbers indicate unclear perception.

Continuous presentation

Filename: AlbertetalCon2013.RData with **variables**

- `daten.2013con1`, `daten.2013con2`: Lists with 105 elements each being a data frame with the start and end of the experiment in the first column, the pressed button as second column and the time of button presses as third column (unit: millisecond). Attention: Subjects may have pressed the same button subsequently.
- `dom.2013con1`, `dom.2013con2`: Lists with 105 elements each being a vector of the dominance times (in seconds) of the corresponding subject
- `person2013`: vector containing the number of the subjects with data in at least one trial

Last update: 24.07.2017 by Stefan Albert
